# MicroRNA-495 Modulates Neuronal Layer Fate Determination by Targeting *Tcf4*

**DOI:** 10.7150/ijbs.94739

**Published:** 2024-11-11

**Authors:** Yunli Pang, Xiangbin Ruan, Wei Liu, Lin Hou, Bin Yin, Pengcheng Shu, Xiaozhong Peng

**Affiliations:** 1 State Key Laboratory of Common Mechanism Research for Major Diseases, Department of Biochemistry & Molecular Biology, Medical Primate Research Center, Neuroscience Center, Institute of Basic Medical Sciences Chinese Academy of Medical Sciences, School of Basic Medicine Peking Union Medical College, Beijing 100005, China.; 2 State Key Laboratory of Respiratory Health and Multimorbidity, Beijing 100005, China.; 3 Institute of Laboratory Animal Science, Chinese Academy of Medical Sciences & Peking Union Medical College, Beijing 100021, China.

**Keywords:** miR-495-3p, neuronal layer, fate determination, TCF4, mouse cerebral cortex.

## Abstract

During cortical development, the differentiation potential of neural progenitor cells (NPCs) is one of the most critical steps in normal cortical formation and function. Defects in this process can lead to many brain disorders. MicroRNA dysregulation in the dorsolateral prefrontal cortex is associated with risk for a variety of developmental and psychiatric conditions. However, the molecular mechanisms underlying this process remain largely unknown. In this study, we found that microRNA-495-3p (miR-495) is expressed in NPCs of the developing mouse cerebral cortex. Furthermore, aberrant expression of miR-495 promotes the formation of superficial neurons. Our results suggest that miR-495 can target transcription factor 4 (TCF4), a gene linked to the neurodevelopmental disorder Pitt-Hopkins syndrome (PTHS), to ensure normal differentiation of NPCs in the developing cerebral cortex. Furthermore, TCF4 loss-of-function and gain-of-function experiments show opposite effects on miR-495 regulation of neural progenitor differentiation potential. Together, these results demonstrated that miR-495 regulates cortical development through TCF4 for the first time.

## Introduction

The neocortex of the mammalian brain is one of the most powerful and structurally complex tissues in the human body, characterized by a six-layer organization and composed of thousands of cell types with different morphologies and physiological functions. During the development of the neocortex, the pseudostratified neuroepithelium cells located in the ventricular zone (VZ) produce radial glial cells (RGCs)^1^. RGCs self-renew symmetrically to generate two neural progenitors and divide asymmetrically to produce a neuron and a neural progenitor, or they indirectly yield intermediate progenitors (IPs) in the SVZ (sub-ventricular zone, SVZ)^2^. RGCs directly and indirectly generate projection neurons in layers Ⅵ, Ⅴ, Ⅳ, Ⅲ, and Ⅱ of the neocortex in order from E12.5 to E16.5, in which the later-generated cortical plate cells migrate across early-born neurons to settle into superficial layers, creating the characteristic "inside-out" organization of the neocortex^3^,^4^. Abnormal neurogenesis and layer formation have been linked to various pathologies, such as intellectual disability, language deficits, attention deficit hyperactivity disorder (ADHD), and autism spectrum disorders (ASDs)^5^-^7^. In addition, a study found that symptoms associated with autism can occur in transgenic mice with increased numbers of superficial neurons^8^. The molecular mechanisms underlying abnormal neurogenesis and layer formation have been a hot topic and challenge in developmental neuroscience research.

MicroRNAs (miRNAs) are a class of small noncoding RNAs of approximately 23 nucleotides that were first identified by Lee *et al.* in nematodes^9^, and they act primarily by targeting the mRNA 3'UTR of target genes to inhibit their translation and cause their degradation^10^. Data obtained in previous studies^11^ using conditional mouse strains carrying a deletion of *Dicer*, a key gene for miRNA maturation, indicated that *Dicer*-deficient mouse embryos show cortical malformations.

Recently, our laboratory found that temporal gradients of miRNA expression in the developing neocortex are essential for timing neocortical layer formation and laminar fates. MiR-128 selectively specifies Layer Ⅵ neurons, miR-9 specifies Layer Ⅵ neurons, and let-7 defines Layers Ⅳ, Ⅲ, and Ⅱ during neocortical neurogenesis. Let-7 targets *Hmga2* to regulate the molecular program by which temporal miRNAs modulate a SOX5-to-CUX1 program for neocortical layer formation^12^. This study identified miR-495, a conserved miRNA in various species, including humans, mice, rats, pigs, cows, sheep, and chickens, as a cortical neuron fate-determination regulator. MiR-495 (Gene ID: 751522) is located on mouse genome chromosome 12 (Chr12qF1) and belongs to the *Dlk1-Dio3* miRNA cluster. MiR-495 was reported as a brain-specific miRNA involved in forming a network of microRNA‒gene interactions in neurodevelopment^13^ and is associated with several neurodevelopmental disorders, especially schizophrenia (SCZ) and obsessive-compulsive disorder (OCD)^14^. During non-small cell lung cancer (NSCLC), miR-495 targets *Tcf7l2*, and inactivates the Wnt/β-catenin pathway to inhibit progression^15^. However, the role of miR-495 in cortical development remains unclear.

To understand the role of miR-495 in neocortex development, we first examined the spatial and temporal expression pattern of miR-495 in developing mouse brains. We constructed recombinant plasmids to knock down and overexpress miR-495 and used *in utero* electroporation (IUE) technology to achieve the abnormal expression of miR-495 *in vivo*. We analysed whether miR-495 affects the timing of cortical layer formation and laminar fates. To do this, we conducted an analysis of immunofluorescence staining using antibodies for cortical layer markers. We then identified five candidate target genes of miR-495 using a dual-luciferase reporter assay, including *Pak3, E2f2, Tcf4, Pbrm1, and Zfp36l1*. Finally, we analysed the spatial and temporal expression patterns of these target genes. Tcf4 was chosen as the target gene for further investigation due to its expression pattern and biological functions. We used the IUE method to knock down and overexpress Tcf4 with recombinant plasmids. We conducted immunofluorescence staining analysis using cortical layer markers to determine whether abnormal TCF4 expression affects the timing of neocortical layer formation and laminar fates.

## Results

### miR-495 is expressed in the germinal zone and cortical plate of the developing cerebral cortex

**Figure 1:**
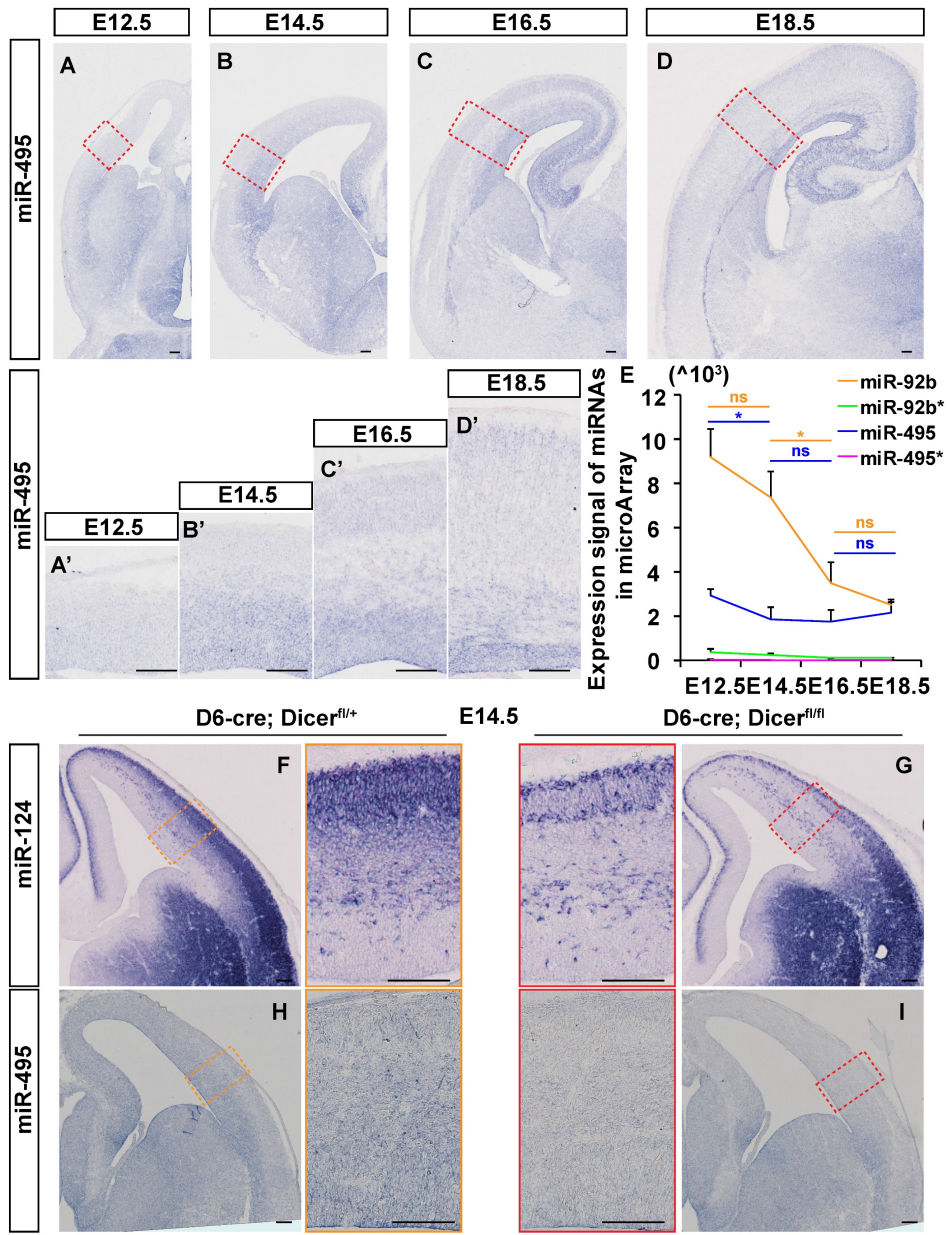
** miR-495 expressed in dorsolateral areas of the neocortex. (A- D)** The expression patterns of miR-495 were detected by *in situ* hybridization on coronal sections of E12.5, E14.5, E16.5, and E18.5 embryonic telencephalons. Scale bar: 100 µm. **(A'-D')** The expression patterns of miR-495 in dorsolateral areas of the neocortex at E12.5, E14.5, E16.5, and E18.5 were detected (positions shown in the red dashed boxes corresponding to the same developmental stages of **(A-D)**. Scale bar: 100 µm. **(E)** RNA samples were obtained from embryonic dorsal forebrains at various stages (from E12.5 to E18.5) in order to detect miR-495 expression. The miR-495* represents mmu-miR-495-5p, while miR-495 represents mmu-miR-495-3p. The following miRNAs were used as controls: miR-495*, miR-92b* (miR-92b-5p) and miR-92b (miR-92b-3p). Each experiment was performed in triplicate, and data are presented as mean ± SD. Statistical significance was determined using an unpaired two-tailed Student' s t-test. The statistically significant P values are shown as *P<0.05. **(F-I)** The expression patterns of miR-124 **(F and G)** and miR-495 **(H and I)** were detected by *in situ* hybridization on coronal sections of E14.5 embryonic telencephalons in *Dicer* cKO mice. Scale bar: 100 µm.

MiR-495 was observed to increase overall in the embryonic brain from E12.5 to E18.5 by *in situ* hybridization (ISH) method (Fig. 1A-1D). In order to ascertain the efficacy of the proposed methodology, a scrambled probe is employed as a negative control in the same condition (Fig. S1 I and J). MiR-495 was highly expressed in the pallial-subpallial boundary (PSB) at E12.5 and in the germinal zone (GZ) at E14.5 (Fig. 1A', 1B'). At both E16.5 and E18.5, miR-495 was also highly expressed in the ventricular GZ and cortex plate (CP) (Fig. 1C', 1D'). Microarray analysis was used to examine the expression signal of miR-495, miR-495*, miR-92b, and miR-92b*. It was found that miR-495* and miR-92b* were rarely expressed in the developing cerebral cortex. The expression of miR-495 decreased from E12.5 to E14.5 (p < 0.05), while the expression of miR-92b decreased from E14.5 to E16.5 (p < 0.05) (Fig. 1E). A reduction in miR-124-3p (miR-124), was identified in the cerebral cortex of *Dicer* knockout mice (Fig. 1F-1G), confirming the authenticity of the miRNA *in situ* hybridization signal. Similarly, miR-495 was rarely expressed in the cerebral cortex of *Dicer* knockout mice (Fig. 1H-1I).

### miR-495 has an independent expression pattern in the *Dlk1-Dio3* miRNA cluster

**Figure 2:**
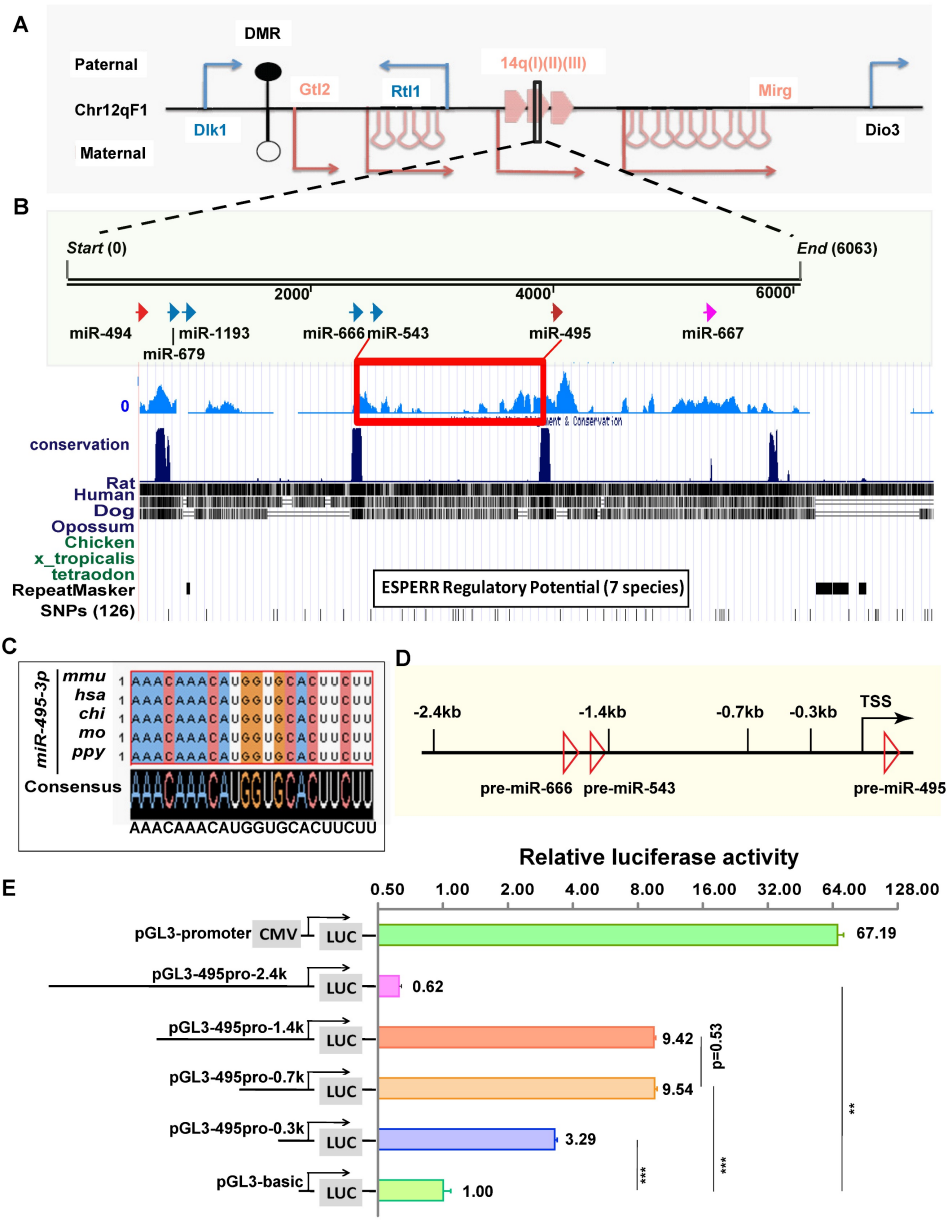
** miR-495 has a relatively independent transcriptional pattern in the *Dlk1-Dio3* region. (A)** The relative positions of miR-495 in the *Dlk1-Dio3* region are shown. Blue genes (*Dlk1, Rtl1*, and *Dio3*) and arrows indicate paternal genes, and red genes (*Gtl2* and *Mirg*) and arrows indicate maternal genes. DMR is the "differentially methylated region." The miRNAs adjacent to miR-495 in the genome are, from left to right, miR-494, miR-679, miR-1193, miR-666, miR-543, and miR-667 downstream. **(B)** Species evolutionary conservation of miR-495 was analysed. **(C)** The mature sequence of miR-495 was compared in various mammals. **(D)** The schematic diagram depicts the genome within 2.4 kb upstream of miR-495. **(E)** The luciferase reporter assay identified the promoter region of miR-495. Sequences of different lengths upstream of pre-miR-495 were inserted into the pGL3 vector, where pGL3-promoter (CMV) was used as a positive control. The corresponding vectors' relative proportions of luciferase activity are shown on the right. Each experiment was performed in triplicate, and data are presented as mean ± SD. Statistical significance was determined using an unpaired two-tailed Student's t-test. P values are shown as ** P<0.01 and ***P<0.001.

The genomic locus of miR-495 is located in the Dlk1-Dio3 miRNA cluster at the distal end of mouse chromosome 12 (Chr12qF1), where more than 40 miRNA loci are distributed in this genomic region (Fig. 2A). To analyse the evolutionary conservativeness of miR-495, we used DNAMAN software to align the sequences from miR-543 to miR-495 among seven species (Fig. 2B), and the findings suggested that the sequence has conserved transcriptional regulatory elements. In addition, the mature sequence of mouse miR-495 (mmu-miR-495-3p, similarly hereinafter) is identical to that of several mammals, such as humans, orangutans, rats, and pigs (Fig. 2C), indicating that miR-495 from diverse species may have conserved functions. To identify the promoter region of miR-495, we inserted sequences of different lengths upstream of the pre-miR-495 genome, including -2.4 kb, -1.4 kb, -0.7 kb, and -0.3 kb, into the pGL3-basic vector with the 5' end of pre-miR-495 as the start site (Fig. 2D), and we used the Dual Luciferase Reporter Assay System to verify whether these sequences were transcriptionally active. pGL3-basic and pGL3-495pro-2.4k (including premiR-666 and premiR-543) showed very little apparent transcriptional activity, with that of pGL3-495pro-2.4k being slightly decreased compared with pGL3-basic (0.62:1, p=0.0014). Both pGL3-495pro-1.4k and pGL3-495pro-0.7k had apparent transcriptional activity, and there was no significant difference between them (9.42:1 and 9.54: 1, p<0.001, p=0.53), indicating that there is no essential transcriptional activity regulatory element between 1.4 kb and 0.6 kb upstream of miR-495. In comparison, pGL3-495pro-0.3k had slight transcriptional activity (3.29:1, p<0.001), differing from pGL3-495pro-0.6k and pGL3-495pro-1.4k (p<0.001) (Fig. 2E). Thus, miR-495 has an independent transcriptional pattern in the *Dlk1-Dio3* miRNA cluster.

We detected the expression patterns of miR-494 and miR-667 close to miR-495 by *in situ* hybridization on coronal sections of the embryo from E12.5 to E18.5. The *in situ* hybridization results showed that miR-494 and miR-667 were expressed in the cerebral cortex (Fig. S1A-H). At the E14.5 developmental stage, the expression of miR-494 gradually decreased from E14.5 to 18.5 (p < 0.05; p < 0.01). In contrast, miR-667 showed a transient decrease from E12.5 to 14.5 (p < 0.05) and then maintained a consistently lower level of expression (Fig. S1K). Therefore, the expression pattern of miR-495 differs from those of miR-494 and miR-667 in the Dlk1-Dio3 miRNA cluster. Therefore, the expression pattern of miR-495 differs from those of miR-494 and miR-667 in the Dlk1-Dio3 miRNA cluster.

### MiR-495 promoted early generation of upper-layer cortical neurons

**Figure 3:**
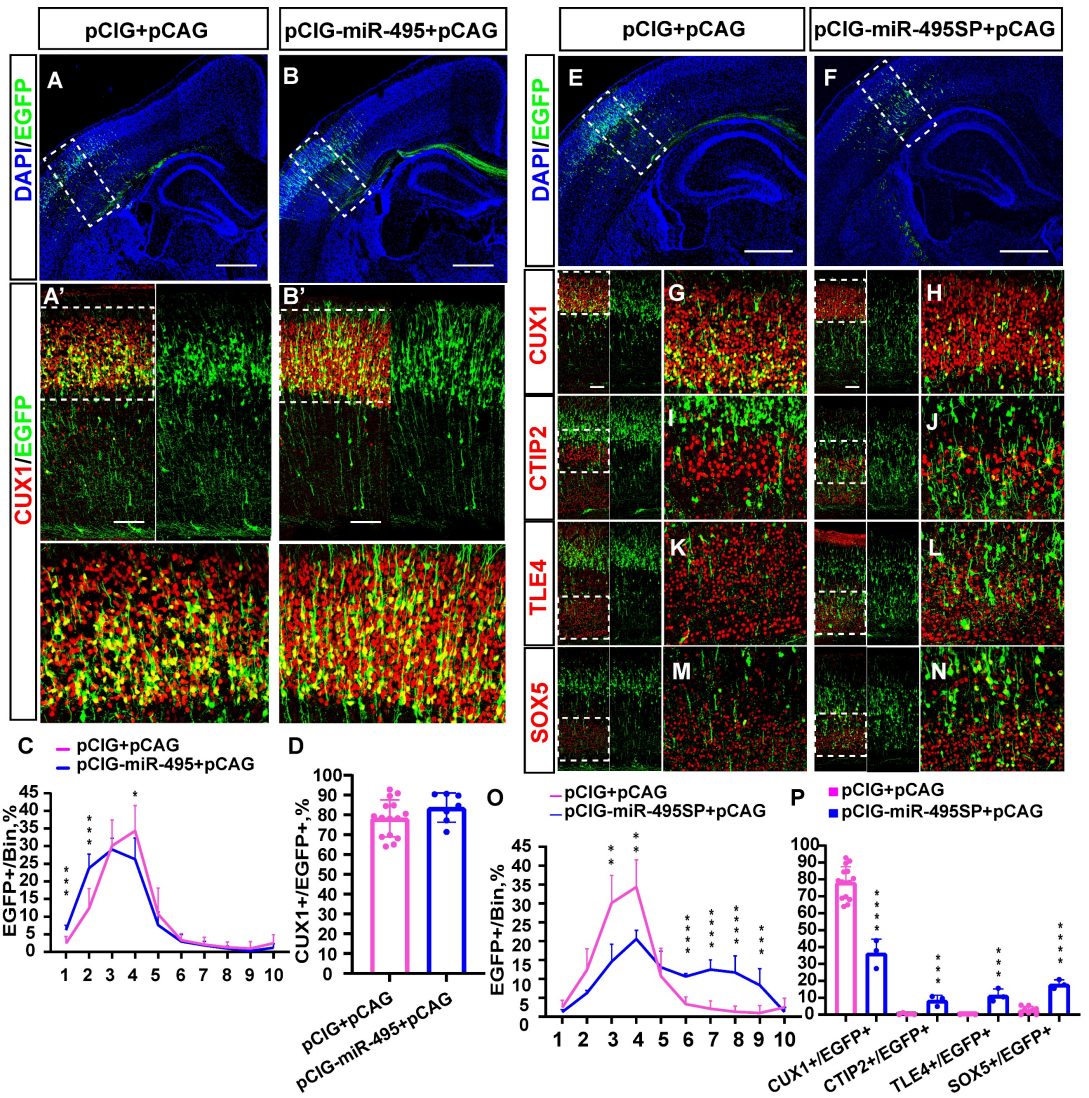
** miR-495 regulates neuronal layer fate determination in the neocortex. (A-B)** Overexpression of miR-495 promotes the generation of superficial neurons. pCIG+pCAG (control) or pCIG-miR-495+pCAG (miR-495) were electroporated into the embryonic mouse brain at E13.5. **(A'-B')** The P3 brain sections were immunostained with anti-Cux1 antibody. (A'-B') indicates the area within the dashed box **(A-B)**. The white dashed box of **(A'-B')** delineates the area of neurons in the upper cortex. Scale bar: 500 µm **(A-B)** and 100 µm **(A'-B') (C)** The sections labelled A' to B' were divided into 10 bins to count the distribution of EGFP-positive cells in the cortex. These sections were derived from the electroporated mouse brains, which were from different littermates (control: n=15 sections from 13 brains, miR-495: n=7 sections from 5 brains)**. (D)** Quantification of Cux1 and EGFP co-labelled cells from the sections of P3 embryos brains from different littermates of E13.5-P3 electroporation. (control: n=15 sections from 13 brains, miR-495: n=7 sections from 5 brains) **(E-N)** Electroporation of mouse embryos with pCIG+pCAG and pCIG-miR-495SP+pCAG at E13.5, respectively, on brain sections immunostained with cortical markers (CUX1, CTIP2, TLE4, and SOX5) at P3. The left side of each figure (Scale bar: 100 µm) shows the cortical extent within the solid white boxes in E and F (Scale bar: 500 µm), and the right side is a zoomed-in view of the dashed box on the left. **(O)** The sections labelled G to H were divided into 10 bins to count the distribution of EGFP-positive cells in the cortex. These sections were derived from the electroporated mouse brains, which were from different littermates (control: n=15 sections from 13 brains, miR-495SP: n=3). **(P)** Cells co-labelled with EGFP and CUX1, CTIP2, TLE4, and SOX5 in electroporated sections from different litters of E13.5-P3 embryos were quantitatively analysed (control: n=15 sections from 13 brains, miR-495SP: n=3). Statistical significance was determined using an unpaired two-tailed Student's t-test. Results are expressed as the mean ±SD. *P<0.05, ** P<0.01 and ***P<0.001.

To explore the function of miR-495 in the developing cortex, we first constructed a recombinant plasmid with miR-495 overexpression (pCIG-miR-495) and miR-495 inhibition (pCIG-miR-495SP). After successfully verifying the efficiency of the constructs carrying pCIG-miR-495 and pCIG-miR-495SP (Fig. S2), we electroporated the constructs into mouse embryonic brains at E13.5 and then collected and stained the brain sections at P3. The staining results (Fig. 3A, 3A') of normal control group showed that most of the neurons with EGFP expression were distributed in layer Ⅳ, with a few in layer Ⅱ/Ⅲ in the cerebral cortex (Fig. 3C pCIG+pCAG: bin4: 34.3%, bin3: 30.1%, bin2: 12.4%, and bin1: 2.4%). In contrast, more neurons expressing miR-495 localized in the superficial cortical layers (Fig. 3B, 3B'), mainly in layer Ⅱ/Ⅲ/Ⅳ (Fig. 3C pCIG+pCAG: pCIG-miR-495+pCAG, bin1: 2.4%: 6.2%, p<0.001; bin2: 12.4%, 23.7%, p<0.001; bin4: 34.3%, 26.3%, p<0.05;). In addition, the proportion of EGFP and CUX1 dual-positive neurons with pCIG-miR-495 increased but was not statistically significant (Fig. 3D pCIG+pCAG: pCIG-miR-495+pCAG, 78.2%: 83.7%, p>0.1).

Next, we electroporated the constructs with miR-495 into the mouse embryonic brain at E13.5 and then collected and stained the brain sections at P9. As shown in Figure S3, we found projection fibres in the contralateral cortex. In contrast, projection fibres were rarely observed in the control group (pCIG+pCAG). These results suggest that the overexpression of miR-495 can promote the premature occurrence of cortical neurons in layer Ⅱ/Ⅲ of the cerebral cortex. As shown in Figure S3, the overexpression of miR-495 promotes the early generation of layer Ⅱ/Ⅲ cortical neurons.

We electroporated the constructs with miR-495SP into the mouse embryonic brain at E13.5. Subsequently, we collected the brain sections and stained them using antibodies against CUX1, CTIP2, TLE4, and SOX5 at P3 (Figure 3E-N. The proportion of superficial cortical EGFP-positive cells was reduced (Fig. 3O, pCIG+pCAG: pCIG-miR-495SP+pCAG bin3: 30.1%, 14.6%, p<0.01; bin4: 34.3%, 20.6%, p<0.01), while the proportion of cells distributed in the deep-layer cortical neurons was significantly increased, mainly in layer Ⅴ/Ⅵ (Fig. 3O, pCIG+pCAG: pCIG-miR-495SP+pCAG, bin6: 3.3%:10.6%, p<0.001; bin7: 2.1%, 12.5%, p<0.001; bin8: 1.3%, 11.7%, p<0.001; bin9: 1.0%, 8.3%, p<0.001). The proportion of CUX1-positive cells carrying miR-495SP decreased (Fig. 3P, pCIG+pCAG: pCIG-miR-495SP+pCAG, 78.2%, 36.3%, p<0.001). The proportion of deep-layer neurons, such as SOX5-positive, CTIP2-positive, and TLE4-positive cells carrying miR-495SP, decreased (CTIP2/EGFP: pCIG+pCAG 0.3%**,** pCIG-miR-495SP+pCAG 8.4%**,** p <0.001; TLE4/EGFP: pCIG+pCAG 0.2%, pCIG-miR-495SP+pCAG 11.4%, p<0.001; SOX5/EGFP: pCIG+pCAG 2.7%, pCIG-miR-495SP+pCAG 18.0%, p <0.001). Similarly, the knockdown of miR-495 by antagomiR-495 also inhibits the superficial layer neuron generation and promotes the deep-layer neuron generation. (Fig. S4).

### The transcription factor 4 gene, *Tcf4*, is a direct target of miR-495 in the developing cortex

**Figure 4:**
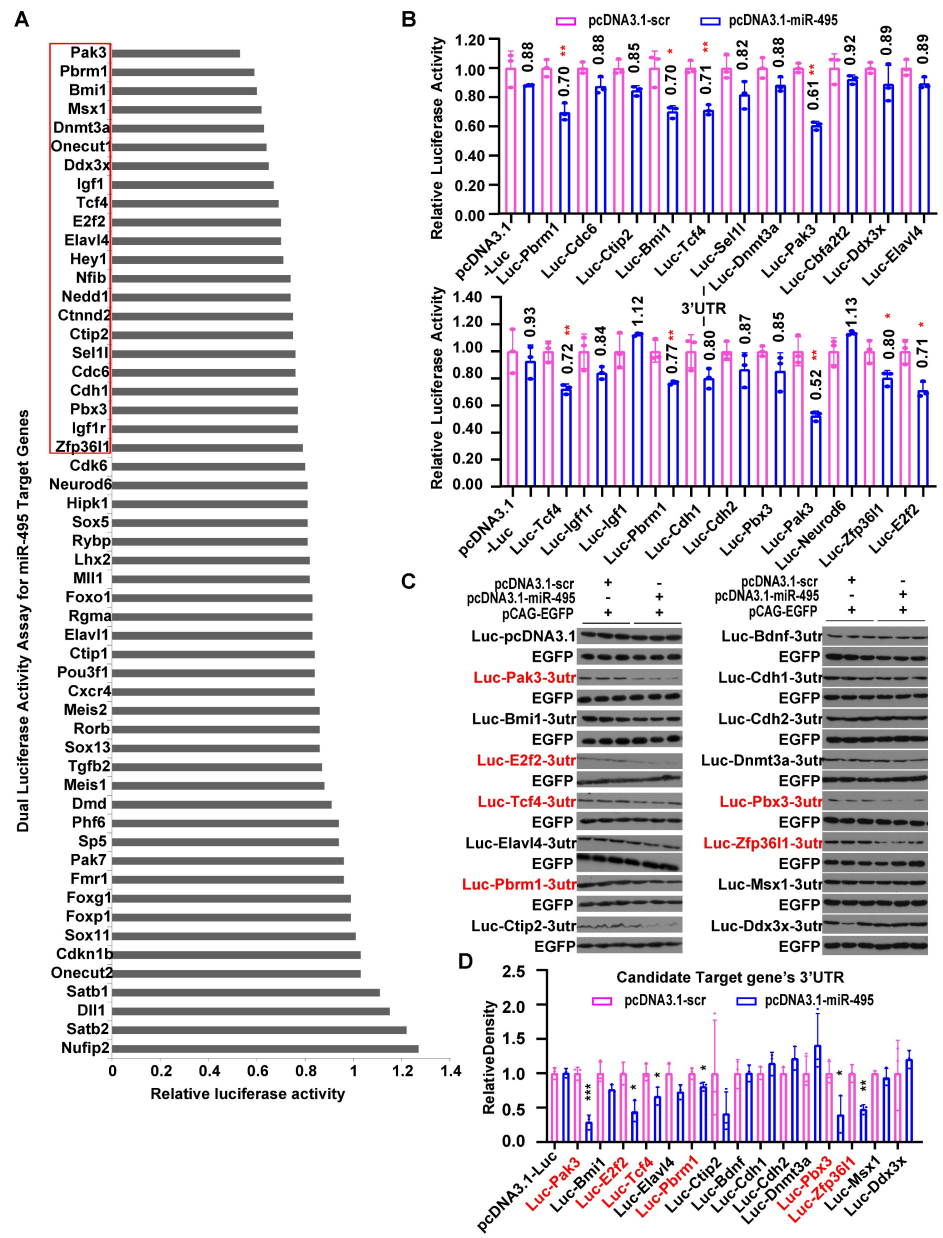
** Screening of miR-495 target genes by the Dual-Luciferase Reporter Gene Assay. (A-B)** Screening of candidate target genes for miR-495 by the Dual-Luciferase Reporter Gene Assay. **(C-D)** Further screening of miR-495 target genes by luciferase immunoblotting. The genes in red are candidate target genes downregulated by miR-495, and the statistically significant P values are shown as *P<0.05 and ** P<0.01. Each experiment was performed in triplicate, and data are presented as mean ± SD. Statistical significance was determined using an unpaired two-tailed Student's t-test.

To identify regulators for miR-495 that might be responsible for neuronal identities, we first used prediction algorithms including TargetScan to find potential target genes. Then, we found 54 genes (Fig. 4A) expressed in the mouse cerebral cortex using databases, including MGI, Eurexpress, Genepaint, Allen Brain, and NCBI. By cloning the 3'UTRs of these genes and co-expressing them with miR-495, we identified 22 genes as potential candidates, in which the luciferase reporter activity was reduced to below 80%, using pRL-TK as an internal reference for luciferase activity (Fig. 4A, 4B). Furthermore, we applied a similar methodology for the Western blot analysis of the luciferase protein, utilizing pCAG-EGFP as the internal reference in place of pRL-TK. As shown in Figure 4C and D, miR-495 significantly inhibited the luciferase activity of reporters including *Pak3* (p < 0.001), *E2f2* (p < 0.05), *Tcf4* (p < 0.05), *Pbrm1* (p < 0.05), *Pbx3* (p < 0.05), and *Zfp36l1* (p < 0.01). Eventually, we selected 5 genes downregulated by miR-495, including *Pak3*, *E2f2*, *Tcf4*, *Pbrm1*, and *Zfp36l1*, considering the results of the Dual Luciferase Reporter Assay System and Western Blotting of luciferase protein.

**Fig 5:**
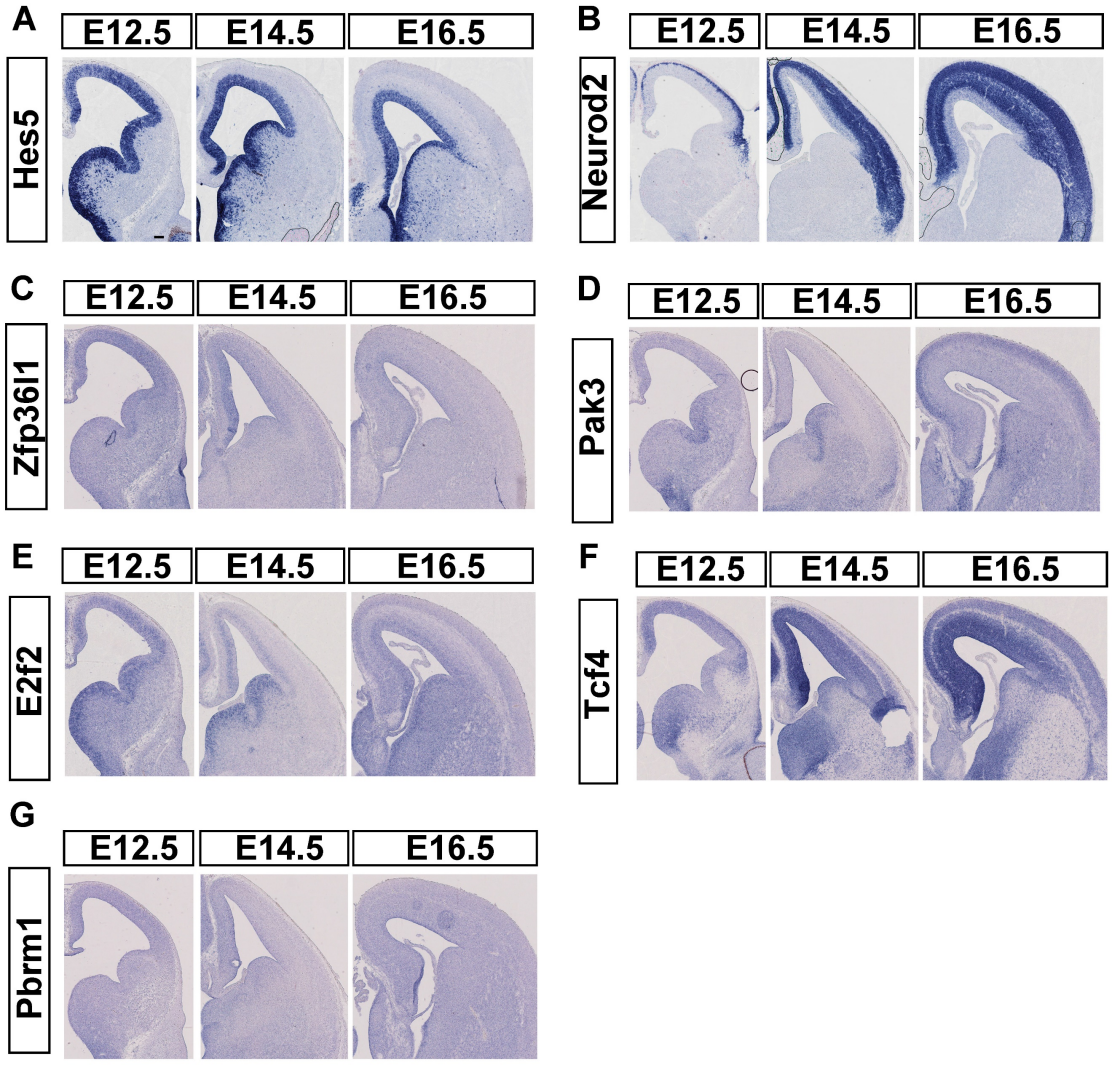
**Expression of candidate target genes of miR-495 in the neocortex of the brain. (A-B)** Positive controls for *in situ* hybridization experiments. **(C-G)** *In situ* hybridization results of candidate target genes. Scale bar: 100 µm.

To compare the mRNA expression pattern of candidate targets to miR-495, we used ISH to detect their spatiotemporal patterns from E12.5 to E16.5. In advance, we selected embryo brain sections from ICR mice to conduct ISH. Initially, we verified Hes5 expression in NPCs and NeuroD2 expression in postmitotic neurons (Fig. 5A, 5B) for use as controls. In our study, GZ highly expressed *Pak3* at E12.5, but CP highly expressed the gene at E16.5. More *Pak3* was expressed in the CP at E16.5, but little *Pak3* was expressed at E14.5 (Fig. 5D). *E2f2* was mainly expressed in the GZ from E12.5 to E16.5, with little expression in the CP at E16.5 (Fig. 5E). *Pbrm1*, with a low expression level, was detected throughout the cortex in the developing mouse neocortex (Fig. 5G). *Zfp36l1* (Fig. 5C) was highly expressed in the cerebral cortex only at E12.5 but was hardly expressed at E14.5 and E16.5. At E12.5, TCF4 exhibited high expression levels, which persisted at E14.5, E16.5, and E18.5 in both the GZ and the cortical plate (Fig. 5F). Tcf4 was detected throughout the developing cortex, with a decreasing trend in the dorsomedial to dorsolateral prefrontal cortex. This pattern contrasts with the miR-495 expression pattern in the GZ.

### TCF4 overexpression increased the generation of deep-layer neurons as an miR-495 target

**Fig 6:**
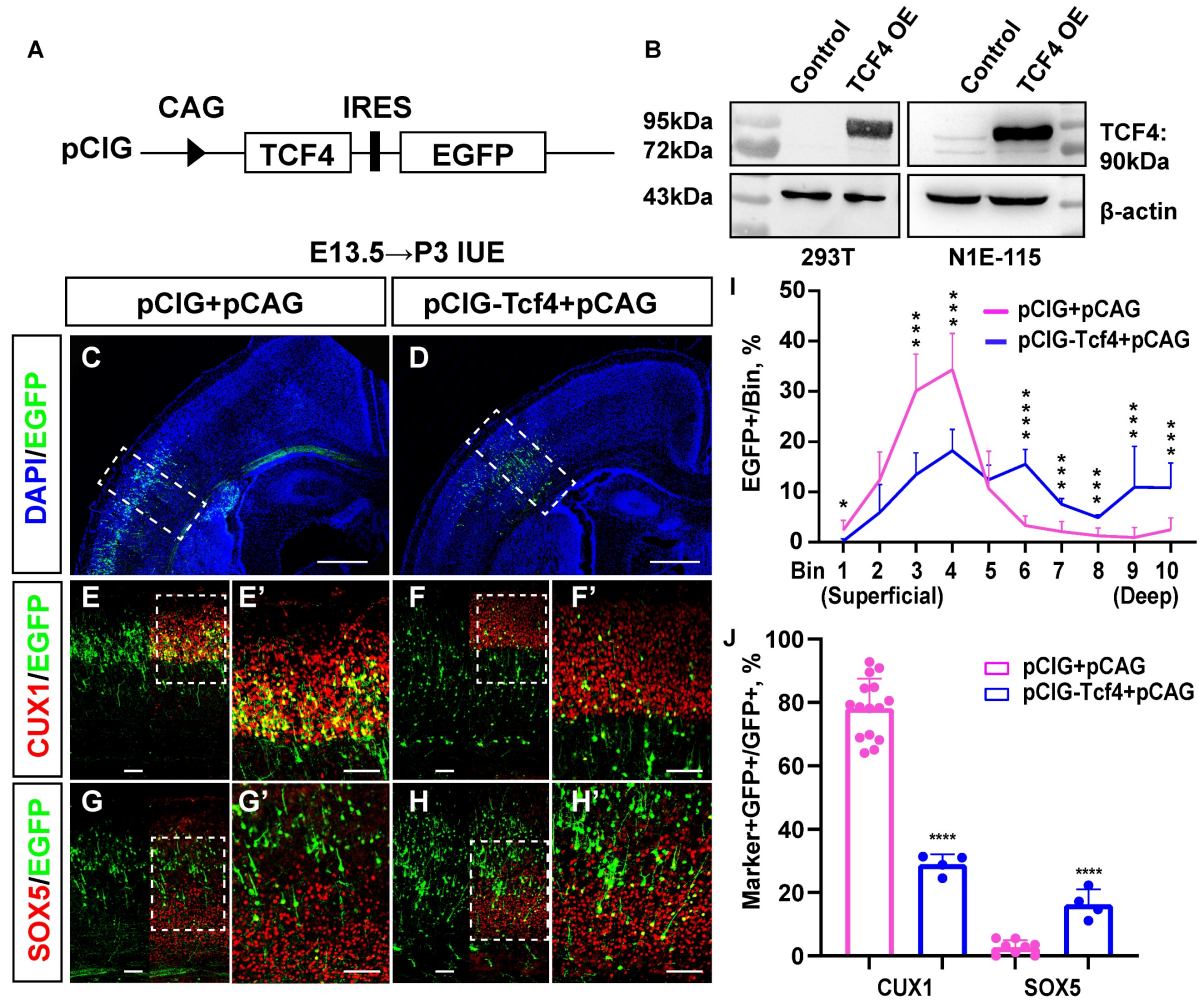
**TCF4 promotes the increased generation of deep-layer neurons. (A)** Schematic diagram of TCF4 overexpression clone construction. **(B)** Western blot validation of TCF4 overexpression clone efficiency. β-actin is as the reference protein. **(C-H)** pCIG+pCAG (control) and pCIG-*Tcf4*+pCAG (TCF4) were electroporated into embryonic brains from mice at E13.5. Brain sections at P3 were immunostained with cortical markers (CUX1 and SOX5). Scale bar: 500 µm **(C, D)** and 100 µm (E-H, E'-H'). **(I)** The sections labelled A' to B' were divided into 10 bins to count the distribution of EGFP-positive cells in the cortex. These sections were derived from the electroporated mouse brains, which were from different littermates (control: n=15 sections from 13 brains, TCF4: n=4). **(J)** Statistical analysis of the percentage of CUX1+/GFP+ cells in **(E', F')** and SOX5+/GFP+ cells in **(G', H')** (control: n**=**15 sections from 13 brains, TCF4: n=4). Statistical significance was determined using an unpaired two-tailed Student's t-test. *P<0.05, ** P<0.01 and ***P<0.001

We first constructed a TCF4 overexpression clone, named pCIG-*Tcf4*, and successfully verified its efficiency (Fig. 6A, 6B). Then, we used the *in utero* electroporation (IUE) method to obtain mouse brain sections from P3 and immunostained them (Fig. 6C-H) for cortical layer markers, including CUX1 (layer Ⅱ/Ⅲ, Ⅳ) and SOX5 (layer Ⅴ/Ⅵ). The immunostaining results showed that more EGFP-positive cells were located in the deep layer of the cerebral cortex (layer Ⅴ/Ⅵ) in the pCIG-*Tcf4* group (Fig. 6I bin 6: 3.3% in the pCIG+pCAG group, 15.5% in the pCIG-*Tcf4*+pCAG group, p <0.001; bin 7: 2.1% in the pCIG+pCAG group, 7.6% in the pCIG-*Tcf4*+pCAG group, p <0.001; bin 8: 1.3% in the pCIG+pCAG group,4.9% in the pCIG-*Tcf4*+pCAG group, p <0.001; bin 9: 1.0% in the pCIG+pCAG group, 10.9% in the pCIG-*Tcf4*+pCAG group, p <0.001; bin 10: 2.5% in the pCIG+pCAG group, 10.8% in the pCIG-*Tcf4*+pCAG group, p <0.001). In comparison, the number of EGFP-positive cells in layer Ⅳ decreased (Fig. 6I bin 3: 30.1% in the pCIG+pCAG group, 13.4% in the pCIG-*Tcf4*+pCAG group, p <0.001; bin 4: 34.3% in the pCIG+pCAG group, 18.3% in the pCIG-*Tcf4*+pCAG group, p <0.001). Compared with that in the pCIG+pCAG group, the proportion of cells co-labelled with CUX1/EGFP was reduced in the pCIG-*Tcf4*+pCAG group (Fig. 6J; 78.2% in the pCIG+pCAG group, 28.9% in the pCIG-*Tcf4*+pCAG group, p <0.001). However, SOX5/EGFP co-labelled cells increased (Fig. 6J 2.7% in the pCIG+pCAG, 16.3% in the pCIG-*Tcf4*+pCAG, p <0.001). Thus, the overexpression of TCF4 in the developing mouse cerebral cortex probably promotes deep-layer cortical neuron generation.

### TCF4 knockdown promoted the early generation of superficial layer neurons

**Fig 7:**
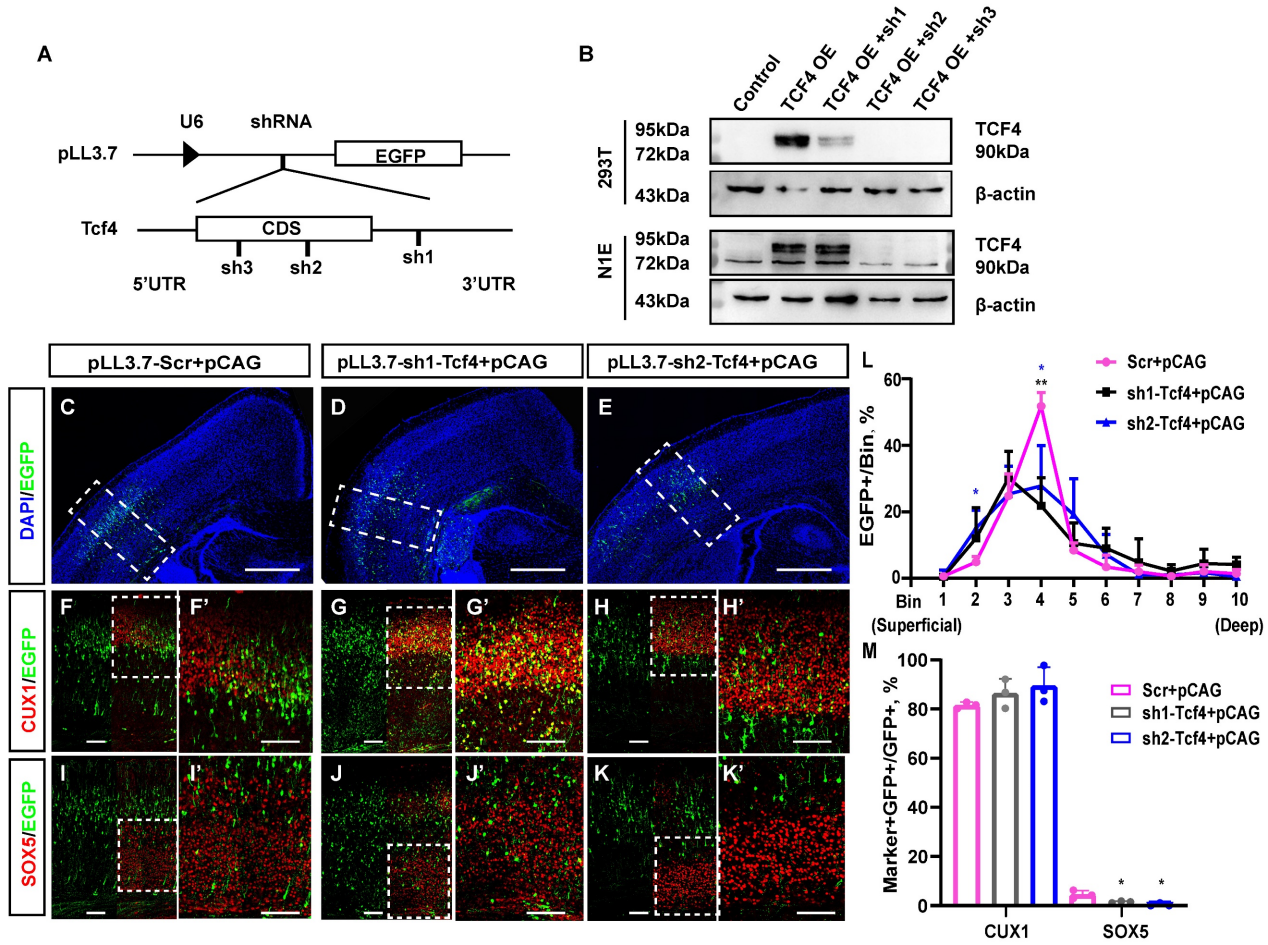
**TCF4 knockdown restrains the generation of deep-layer neurons. (A)** Schematic diagram of TCF4 knockdown clone construction. The designations sh1, sh2, and sh3 refer to pLL3.7-sh1-*Tcf4*, pLL3.7-sh2-*Tcf4*, and pLL3.7-sh3-*Tcf4*, respectively. **(B)** WB validation of TCF4 knockdown clone efficiency. β-actin is as the reference protein. **(C-K)** pLL3.7-Scr+pCAG, pLL3.7-sh1-*Tcf4* + pCAG and pLL3.7-sh2-*Tcf4* + pCAG were electroporated into the embryonic brains of mice at E13.5. Brain sections at P3 were immunostained with cortical markers (CUX1 and SOX5). Scale bar: 500 µm **(C, D, E)** and 100 µm **(F-K, F'-K')**. **(L)** The sections labelled F to H were divided into 10 bins to count the distribution of EGFP-positive cells in the cortex. These sections were derived from the electroporated mouse brains, which were from different littermates (Scr: n=3, sh1-Tcf4: n=3, sh2-Tcf4: n=3). **(M)** The percentage of CUX1+/GFP+ cells and SOX5+/GFP+ cells in **(F-K)** (Scr: n=3, sh1-Tcf4: n=3, sh2-Tcf4: n=3). Statistical significance was determined using an unpaired two-tailed Student's t-test. Results are expressed as the mean ±SD. P values are shown as *P<0.05, ** P<0.01.

We first constructed three knockdown clones of TCF4 using shRNA, named pLL3.7-sh1-*Tcf4*, pLL3.7-sh2-*Tcf4*, and pLL3.7-sh3-*Tcf4*, and successfully verified their efficiency (Fig. 7A, 7B). pLL3.7-sh1-*Tcf4* targets the 3' UTR of *Tcf4*, whereas pLL3.7-sh2-*Tcf4* and pLL3.7-sh3-*Tcf4* target the coding sequence (CDS) of *Tcf4*. Then, we used the same method to analyse whether TCF4 knockdown affects neuronal layer identity with the cortical layer markers CUX1 and SOX5 (Fig. 7C-K'). The immunostaining results illustrated that as many as 15.2% of EGFP-positive cells with pLL3.7-sh2-*Tcf4* were distributed in the superficial cortex layers, while as few as 4.9% of EGFP-positive cells with pLL3.7-Scr were distributed in these layers (Fig. 7L bin 2: 4.9% in pLL3.7-Scr, 15.2% in pLL3.7-sh2-*Tcf4*+pCAG, p <0.05). Moreover, the cells distributed in bin 4 were significantly reduced (Fig. 7L bin 4: 51.7% in pLL3.7-Scr+pCAG, 27.8% in pLL3.7-sh2-*Tcf4*+pCAG, p <0.05). In contrast, 21.8% of EGFP-positive cells with pLL3.7-sh1-Tcf4 were distributed in bin 4, which was significantly lower than that observed in the control (Fig. 7L bin 4: 51.7% in pLL3.7-Scr+pCAG, 21.8% in pLL3.7-sh1-*Tcf4*+pCAG, p <0.01).

In addition, only 0.4% of the EGFP-positive cells with pLL3.7-sh2-Tcf4 expressed SOX5 (Fig. 7M). This was significantly lower compared to the 5.1% observed in the combined pLL3.7-Scr group and pCAG group (p < 0.05). Similarly, only 1.5% of the EGFP-positive cells with pLL3.7-sh1-Tcf4 expressed SOX5, which was lower than the percentage observed with pLL3.7-scr (p < 0.05). Approximately 89.5% of the cells co-labelled with CUX1/EGFP were in the pLL3.7-sh2-*Tcf4*+pCAG group, compared to 81.6% in the pLL3.7-Scr group + pCAG (Fig. 7M); there was no statistically significant difference (p> 0.1). Meanwhile, 86.4% of EGFP-positive cells with pLL3.7-sh1-Tcf4 were found to express CUX1, with no significant difference observed between the pLL3.7-sh1-Tcf4 group and the pLL3.7-Scr+pCAG group. The overexpression of TCF4 *in vivo* resulted in an increase in deep layer neurons. However, after TCF4 knockdown, the number of EGFP-positive cells in the upper-layer neurons seemed to increase slightly, but there was no statistical difference compared to the control group. Some of these cells were co-labelled with CUX1 (Fig. 7M), which was similar to the phenotype of miR-495 overexpression (Fig. 3B, B').

To observe whether Tcf4 can rescue the cortical developmental abnormalities induced by miR-495, the plasmids pCIG+pCAG and pCIG-miR-495+pCIG-Tcf4+pCAG were co-electroporated into mouse neural progenitor cells at E13.5. Subsequently, sections of the mouse telencephalon were collected at P3 for immunofluorescence staining of CUX1 and SOX5. The results demonstrated that the majority of EGFP-positive cells electroporated with pCIG-miR-495+pCIG-Tcf4+pCAG were able to revert to a normal fate and migrate to the appropriate location (Fig. S5). However, while the majority of EGFP-positive cells were capable of migrating and determining their fates normally, a subset exhibited abnormal behavior. The count of EGFP- and CUX1-positive cells that were co-transfected with pCIG-miR-495+pCIG-Tcf4+pCAG was found to be decreased when compared to those that were transfected with pCIG+pCAG (p < 0.01). Conversely, it was increased when compared to those transfected with pCIG-Tcf4+pCAG (p < 0.001) (Fig. S5H). We first revealed that miR-495 might participate in the determination of neuronal laminar fate in the developing mouse cerebral cortex through its target gene, *Tcf4*.

## Discussion

Benetatos *et al.* reported that miR-495 belongs to the *Dlk1-Dio3* miRNA cluster^16^, which is located on mouse chromosome 12 (Chr12qF1) and contains the paternally expressed imprinted genes *Dlk1*, *Rtl1*, and *Dio3* and the maternally expressed imprinted genes *Gtl2* and *Mirg*. The *Dlk1-Dio3* miRNA cluster includes over 40 miRNA loci, such as miR-494, miR-679, miR-1193, miR-666, miR-543, and miR-667. MiR-495 has been found to exhibit distinct developmental and lamina-specific expression in the human prefrontal cortex (PFC), with expression levels particularly high in neurons positioned in and around layer II^17^. However, previous studies have yet to demonstrate the role of miR-495 in cortical development, with its target genes remaining unclear. This study found that miR-495 is expressed in the germinal zone and cortical plate of the developing cerebral cortex, which indicates that miR-495 may have an essential function during cortical development. We recognized *Tcf4*, a gene mutated in PTHS, as a target gene of miR-495 and verified that a gain of function (GOF) of TCF4 generates increased number of deep-layer neurons. These findings identified a novel miR-495-*Tcf4* regulatory pathway controlling neuron fate determination in the developing neocortex, providing new ideas for the research and treatment of PTHS.

Overexpression of miR-495 increased the number of superficial neurons. Although layer Ⅱ/Ⅲ/Ⅳ cortical neurons expressed CUX1, there were some differences among them. While layer Ⅱ/Ⅲ cortical neurons are conical neurons with axons and project to the contralateral motor cortex or ipsilateral cortex, layer Ⅳ is composed of pyramidal neurons that receive information from the thalamus^18^. The miR-495-overexpressing neurons showed neural projections similar to those of superficial neurons. A possible mechanism by which miR-495 causes "abnormal" neurons in the cortex is that miR-495 knockdown "reverses" the potency of NPCs, allowing them to produce not only primarily superficial layer neurons after E13.5 but also deep layer neurons.

MiR-495 regulates biological functions by suppressing transcription factor levels. A study found that miR-495 is involved in the multilevel regulation of BDNF expression in the developing PFC as a posttranscriptional inhibitor of BDNF^17^. We concentrated on the gene *Tcf4*, as its mutation is a cause of Pitt-Hopkins syndrome, and accumulating evidence suggests that Pitt-Hopkins syndrome is associated with aberrant cortical neurons^19^,^20^. TCF4 is also reported to be a master regulator in schizophrenia through the deconvolution of transcriptional networks^21^.

TCF4 is a transcription factor encoded by the *Tcf4* gene and belongs to the bHLH (basic helix-loop-helix, bHLH) family. TCF4 is widely expressed in cerebral cortex and subcortical structures in developing and adult mice 22. As reported, TCF4 dysfunctional mice exhibit delayed neuronal migration, a significant increase in neurons in the upper cortex, and abnormal dendrite and synapse formation 23. The Tcf4 defect has the dysfunction of neuronal terminal localization and impaired electrophysiological function. Tcf4 controls the localization of neurons during brain development by regulating the expression of cell adhesion molecule Fn1^24^. Aberrant TCF4 expression results in abnormal neuronal differentiation and terminal localization, consistent with our results in mouse embryos. Neurons overexpressing TCF4 are primarily located in the deep layer of the cerebral cortex, while neurons with TCF4 knockdown are mainly found in the superficial layer. However, there is no statistical difference in the percentage of CUX+ cells within the EGFP+ cell population between the control group and the shRNAs-Tcf4 group. The observed phenomenon warrants additional research to uncover the root causes behind it. MiR-495 may regulate nervous system development through a similar pathway. In another word, miR-495 down-regulates TCF4 expression to control neuronal differentiation and terminal localization. Furthermore, in a PTHS model constructed using patient-derived cortical neurons, Brittany A Davis *et al.* 25 discovered that a TCF4 mutation dysregulated RIMBP2, leading to impaired synaptic function. TCF4 plays a crucial role in the development of the cerebral cortex by regulating various functional proteins.

TCF4 can rescue the effect on neuronal layer fate caused by miR-495, but a proportion of cells remained abnormal. This phenomenon may be related to the complexity of miR-495 regulation. MiR-495 is able to regulate multiple target genes, which may be involved in multiple signal pathways and biological processes. Therefore, the role of miR-495 depends on a complex "miRNA-target gene" regulatory network. In this network, miR-495 may interact with different target genes and pathways to affect cell behaviour. In addition, TCF4 alone may not be sufficient to fully rescue the biological effects produced by miR-495. This may be because, although TCF4 is an important transcription factor, it does not completely replace the role of miR-495 in the regulatory network.

Most notably, this is the first study to our knowledge to investigate whether miR-495 regulates the neuron fate determination of the cerebral cortex by targeting *Tcf4*. Our results provide compelling evidence that miR-495 targets *Tcf4* to promote the generation of superficial-layer neurons. Therefore, miR-495-*Tcf4* is a potential treatment target for PTHS. However, some limitations are worth noting. Although our hypotheses were supported, the functions of miR-495's other target genes remain unclear. Future work should aim to find and validate the *in vivo* functions of other target genes that are influenced by miR-495. Furthermore, transgenic mice can be introduced for in-depth study to observe the biological effects of miR-495 and its target genes in a specific region and at a specific time. Promoters that express specificity in terms of time, space, or cell type will be used. It would be valuable to gain a more comprehensive understanding of miR-495's role in neurogenesis.

## Materials and methods

### Plasmids

The premiR-495 amplified from the genomic DNA of adult ICR mice was cloned and inserted into pGEM-T for probe preparation (Promega). The PCR fragments from the candidate target genes of miR-495 were cloned and inserted into pGEM-T for probe preparation (Promega). pT7T3D-PacI was used to construct a plasmid for the probe, which was kindly donated by Prof. Meng-Sheng Qiu of Hangzhou Normal University. Antagomir-495 and antagomiR-NC were obtained from Guangzhou Rui Bo Biotechnology Co.

The complementary sequence of miR-495 was synthesized, annealed and inserted downstream of the luciferase gene in the pcDNA3.1 vector (pcDNA3.1-luc) to construct the miR-495 reporter plasmid. pcDNA3.1(+) was used to construct the miRNA overexpression plasmid pcDNA3.1-luc, which was modified from the vector pcDNA3.1(+) to build the luciferase reporter gene plasmid pGL3-basic. The pGL3-promoter was used to determine miRNA promoter activity (Promega). The full-length 3'UTR of the *Tcf4* gene was generated by total complementary DNA (cDNA) from E14.5 mouse brains and was inserted downstream of the luciferase gene in the pcDNA3.1 vector.

Pre-miR-495 was amplified using genomic DNA from adult ICR mice. It was digested with XhoI and EcoRI and then cloned and inserted into the pCIG vector (provided by Prof. Weimin Zhong's laboratory at Yale University). The pCIG vector was modified by inserting *Bst*EII. Oligonucleotides (sponges) for the binding sites of miR-495 were annealed, ligated, gel-purified, and cloned into the modified pCIG vector digested with BstEII, resulting in the construct labelled miR-495SP.

The oligonucleotide sequences targeting TCF4 were designed and synthesized. The sense oligo format is as follows: 5'T-(GN18) - (TTCAAGAGA)-(81NC)-TTTTTTC. The antisense oligo is the complement of the sense oligo but with additional nucleotides at the 5' end to generate *Xho*I overhang. The above-mentioned oligo sequences were annealed, and inserted into pLL3.7 vector, named pLL3.7-sh1-*Tcf4*. pLL3.7-sh2-*Tcf4*. A scrambling sequence was inserted into pLL3.7 vector as a control**,** named pLL3.7-Scr. The plasmid pCAG-EGFP for *in vivo* electroporation was kindly donated by Prof. Jing Naihe's laboratory, Institute of Cell Biochemistry, Chinese Academy of Sciences. The ORF of the *Tcf4* gene was cloned and inserted into the pCIG vector. The TCF4 knockdown plasmid was constructed using the pLL3.7 vector (Addgene). All sequences used in the clone are given in Table S1.

### Cell culture

HEK-293ET cells (provided by the Cell Centre of Peking Union Medical College) and N1E-115 cells (gifted by Dr. Yan Zhou, Wuhan University) were used. The cells were cultured in complete Dulbecco's modified Eagle medium (DMEM) (Thermo Scientific, Waltham, Massachusetts, USA) with 10% (v/v) foetal bovine serum (FBS). For transfection, plasmids, miR-495 mimics (GenePharma, Shanghai, China) and antagomir-495 (RiboBio, Guangzhou, China) were transfected into HEK-293ET cells using Lipofectamine 3000 (Invitrogen) according to the manufacturer's instructions.

### Dual-luciferase reporter assay

The 3′ UTR of candidate target genes for miR-495 was amplified from mouse cDNA and inserted into the pcDNA3.1-luc vector. Cells were seeded in 24-well plates and transfected with luciferase reporters (pRL-TK, 50 ng/well; firefly luciferase reporter, 200 ng/well) and miRNA mimics (25 nM) after 36 h. To serve as a control, pcDNA3.1-fLuc-3'UTR+pCIG was utilized, while pRL-TK was used as the internal reference for luciferase activity. Then, the cells were collected, and luciferase activity was determined using a Dual-Luciferase Reporter Assay System (Promega, Fitchburg, Wisconsin, USA). The method used for the western blotting of luciferase protein was similar to the one described above. However, the internal reference was changed from pRL-TK to pCAG-EGFP. The expression level of Luciferase was detected using anti-Firefly luciferase antibody, while the expression level of the internal reference was detected using anti-EGFP antibody. The primary antibody was diluted to a concentration of 1:1000.

### Animal

Dr. C.W. in the laboratory provided D6-cre-*Dicer*-floxed mice. Pregnant ICR mice were obtained from the Experimental Animal Centre, Peking University Health Science Centre (Beijing, China). The Institutional Animal Care and Use Committee of the Chinese Academy of Medical Sciences and Peking Union Medical College approved the animal care protocol and experiments. All procedures complied with the Experimental Animal Regulations (China Science and Technology Commission Order No. 2).

### *In situ* hybridization (ISH)

The 4% paraformaldehyde (PFA) in phosphate-buffered saline (PBS) was used to fix mouse embryonic brains, followed by treatment with 25% sucrose in PBS and equilibration in OCT compound (Sakura, San Diego, California, USA). Frozen sections were then incubated with digoxigenin-labelled miRNA probes (Exiqon, Vedbaek, Denmark) and treated according to standard ISH methods^26^. Scramble probe is used as negative control. The probes' primer sequences are shown in Table S1. All probes from microRNA and target genes were purchased from Exiqon. The code number of miR-495 probe is 38609- 05 and the scramble probe sequence is* GCGAUGCUCUAAGGUUCUAUCAA.*

### *In utero* electroporation (IUE)

MiR-495 or its candidate targets were electroporated into pregnant ICR mice at specific times. Pregnant mice at E13.5 were initially anaesthetized with sodium pentobarbital, and the uterine horns were exposed. Electroporation was performed using a BTX-ECM830 electroporator (Harvard Apparatus, Holliston, Massachusetts, USA) with 20-30 V electrical pulses at 950-ms intervals and repeated five times. A 2-3 μg/μL plasmid or 40 μM miRNA anticoagulant spiked with Fast Green (Sigma, Louis, Missouri, USA) was injected into the lateral ventricles of the embryonic brain.

### Immunofluorescence (IF)

Immunofluorescence analysis of brain sections was performed as previously described^26^. DNA staining was performed on sections mounted with coverslips using mounting medium containing DAPI (F6057, Sigma). The primary antibodies used for IF were as follows: PAX6 (prb-278p, Convance), TBR2 (ab23345, Abcam), CUX1 (sc-13024, Santa Cruz), CTIP2 (ab18465, Abcam), TLE4 (sc-9125, Santa Cruz), SOX5 (sc-20091x, Santa Cruz), and EGFP (M20004, Abmart). The secondary antibodies included Alexa Fluor 594 goat-mouse (CA-11005, Molecular), Alexa Fluor 594 goat-rabbit (CA-11012, Molecular), and Alexa Fluor 594 donkey-rat (CA-21206, Molecular). The primary antibody was diluted to a concentration of 1:300 and the fluorescent secondary antibody was diluted to a concentration of 1:800. Images were collected using an Olympus F1000 confocal microscope and processed using the FV10-ASW 3.0 viewer, Adobe Photoshop and Adobe Illustrator. The brain sections at P3 were divided into ten bins using the method described in Figure S5. Bins 1, 2, 3, and 4 corresponded to layers Ⅱ/Ⅲ/Ⅳ, bin 5 and 6 corresponded to layer Ⅴ, and bins 7, 8, 9 and 10 corresponded to layer Ⅵ. We calculated the percentage of EGFP-positive cells in each bin, as well as the percentage of CUX1- or SOX5-positive cells among the EGFP-positive cells.

### Western blot analysis

Total protein was extracted from cultured cells using protein lysis buffer (50 mmol/L Tris, pH 7.5, 150 mmol/L NaCl, 2 mmol/L EDTA and 1% Triton X-100) supplemented with protease inhibitors (Roche, Rotkreuz, Switzerland). Protein concentrations were determined using the BCA Assay Kit (Thermo Scientific™-CN). Protein samples were separated by SDS polyacrylamide gel electrophoresis (PAGE) followed by western blotting using TCF4 antibody (H00006925-M03, Abnova) and β-actin antibody (ab8226, Abcam). Anti-firefly luciferase (ab21176, Abcam) was used to measure dual luciferase reporter gene expression levels. The primary antibody was diluted to a concentration of 1:1000, while the secondary antibody was diluted to a concentration of 1:5000. The β-actin antibody was diluted to a concentration of 1:5000.

### Online databases

Several miRNA target gene prediction databases were used to predict the target genes of miR-495, including Targetscan, miRDB, miRWalk, and miRanda^27^,^28^ (with Targetscan as the primary database). Gene expression profiling databases, including MGI, Eurexpress^29^, Genepaint, and Allen Brain^29^-^32^, were used to determine the level and specificity of expression of candidate target genes in the cerebral cortex. Furthermore, 'GeneRIFs' were also considered. The 'Gene References into Functions' tool in the NCBI database was utilised to search for target genes associated with miR-495 that may regulate neural development.

### Statistical analysis

Data are presented as mean ±s.d. from at least three independent experiments or three individual animals. An unpaired two-tailed Student's t-test was used to compare two groups, unless otherwise stated in the figure legend. Statistical tests were performed using GraphPad Prism 9. P value < 0.05 was considered statistically significant. The statistically significant P values are shown as *P<0.05, ** P<0.01 and ***P<0.001.

## Supplementary Material

Supplementary figures and tables.

## Abbreviations

TCF4: transcription factor 4
miR-495: microRNA-495-3p
PTHS: Pitt-Hopkins syndrome
NPCs: neural progenitor cells
VZ: ventricular zone
SVZ: subventricular zone
IZ: intermediate
GZ: germinal zone
CP: cortex plate
IUE: *in utero* electroporation
ISH: *in situ* hybridization
RGC: radial glia cell
IPs: intermediate progenitors
cDNA: complementary DNA
DAPI: 4'-6-diamidino-2-phenylindole
GFP: green fluorescent protein
RT‒PCR: reverse transcription polymerase chain reaction
PSB: pallial-subpallial boundary
PSB RNA: Ribonucleic Acid in pallial-subpallial boundary
ICR mice: Institute of Cancer Research mice
bHLH: basic helix-loop-helix family
