# Supplementary material for: MicroRNA-495 Modulates Neuronal Layer Fate Determination by Targeting Tcf4
Source: Int J Biol Sci. 2024 Nov 11;20(15):6207–21. doi: 10.7150/ijbs.94739 (PMC11628341; doi:10.7150/ijbs.94739)
Supplement: Supplementary file 1 — Supplementary figures and tables. [file ijbsv20p6207s1.pdf]

Supplementary Information

Journal: International Journal of Biological Sciences

Article Title: MicroRNA-495 modulates neuronal layer fate determination by targeting *Tcf4*

Authors: Yunli Pang[1,5], Xiangbin Ruan[1,5], Wei Liu[1], Lin Hou[1,2], Bin Yin[1,2], Pengcheng Shu[1,2], and Xiaozhong Peng[1,3,4*]

Supplementary Item & Number	Title or Caption
Figure S1	miR-495 has expression patterns different from those of its neighboring miRNAs
Figure S2	Validation of miR-495 overexpression and functional inhibition
Figure S3	Overexpression of miR-495 promoted projection of superficial neurons to the contralateral cortex
Figure S4	Knockdown of miR-495 with antagomiR promotes deep-layer neuron generation
Figure S5	TCF4 rescues miR-495-determined neuronal fate
Table S1	The primer used in the clone
Table S2	The microarray raw data

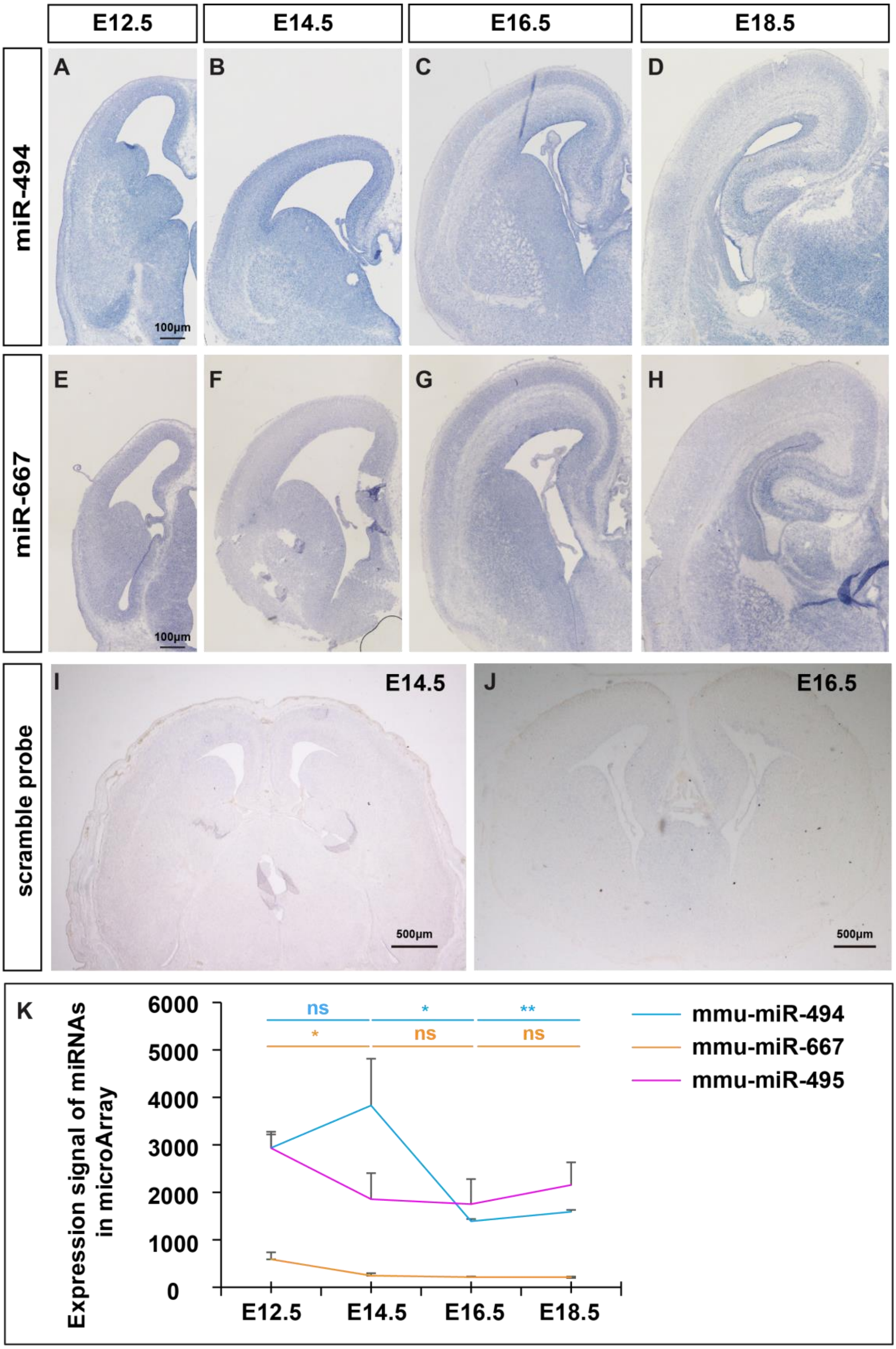

Figure S1 miR-495 has expression patterns different from those of its neighboring miRNAs

(A-D) The expression patterns of miR-494 were detected by in situ hybridization on coronal sections of embryonic telencephalons from E12.5 to E18.5. (E-H) The expression patterns of miR-667 were detected by in situ hybridization on coronal sections of embryonic telencephalons from E12.5 to E18.5. (I-J) The scrambled probe is used as the negative control. (K) The expression patterns of miR-495, miR-494, and miR-667 from E12.5 to E18.5 are shown by microarray data. Each group consists of three replicates, and data are presented as mean ± SD. Statistical significance was determined using an unpaired two-tailed Student's t-test. P values are shown as *P<0.05, ** P<0.01.

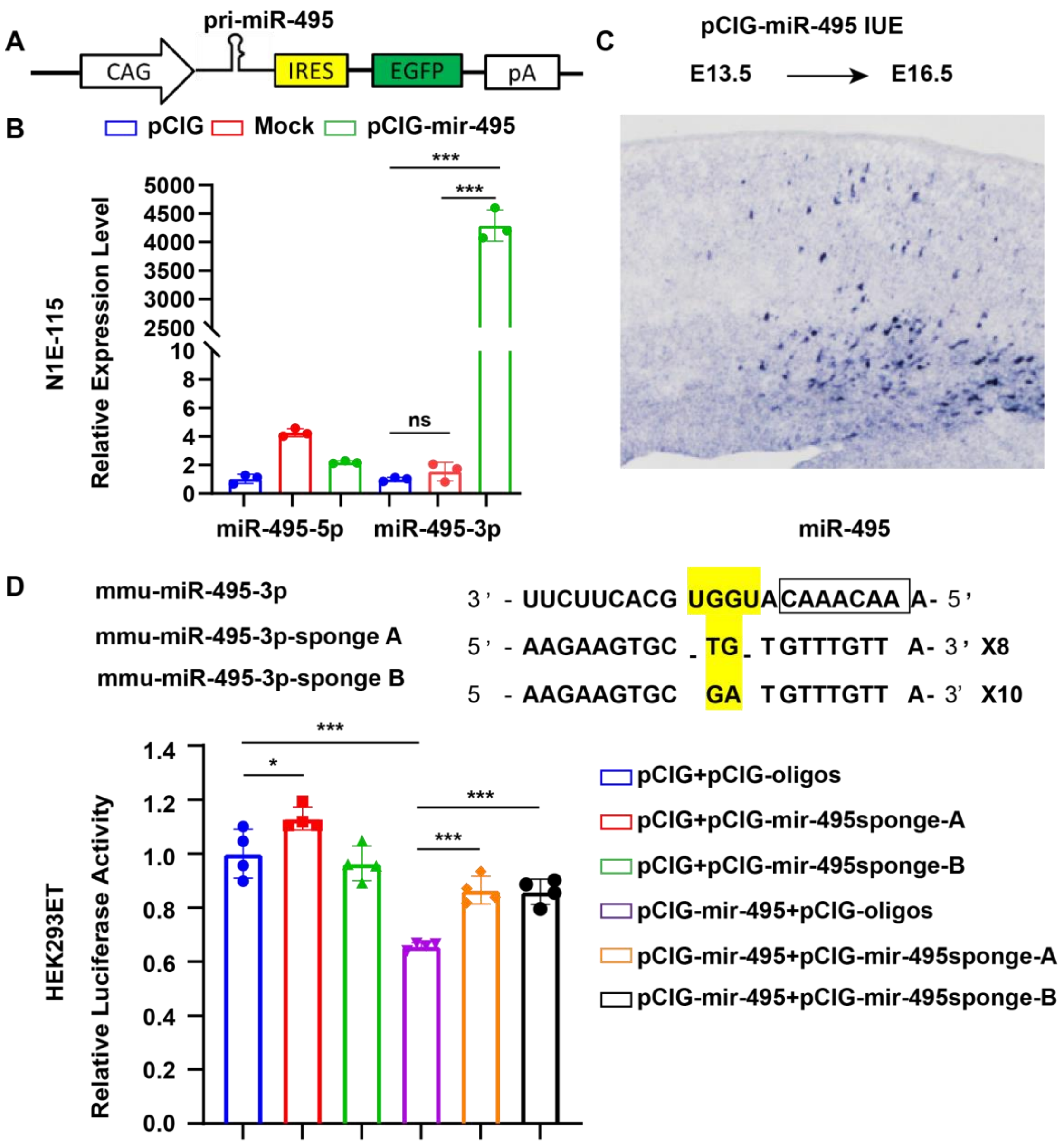

Figure S2 Validation of miR-495 overexpression and functional inhibition

(A) Schematic diagram of pCIG-miR-495 construction. (B) qPCR to measure the levels of miR-495-3p and miR-495-5p in cells transfected with pCIG-miR-495 in vitro. The pCIG group was used as a control group, while the mock group was a blank control group without infection. Each group consists of three replicates. (C) In vivo expression levels of miR-495 in mouse embryos transformed by the pCIG-miR-495 vector were detected by in situ hybridization. (D) Construction and in vitro validation of the miR-495 knockdown vector. A pCIG-miR-495SP knockdown plasmid with a slightly different sequence was constructed. The yellow background indicates the mutated bases, and the box shows the seed sequence

of miR-495 used to identify the 3'UTR of the target gene. The bar graphs show the "rescue" of the target gene Onecut1-3'UTR by the two knockdown vectors or the repression of the overexpressed miR-495. Each group consists of four replicates. The data are presented as mean ± SD. Statistical significance was determined using an unpaired two-tailed Student's t-test. The statistically significant P values are shown as *P <0.05, ** P <0.01 and ***P <0.001.

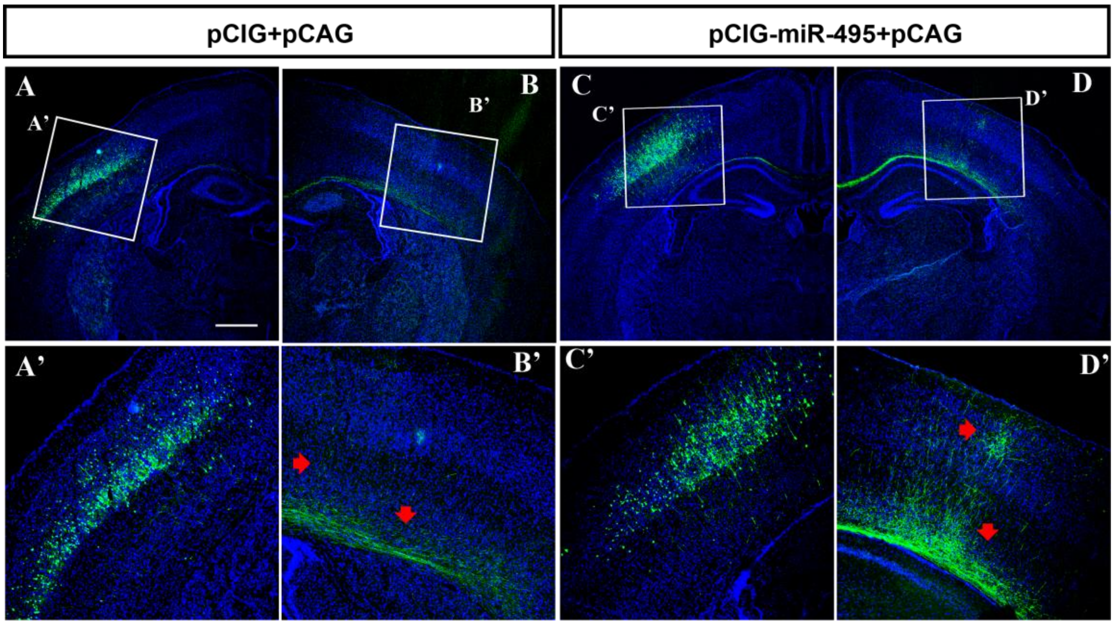

Figure S3 Overexpression of miR-495 promoted projection of superficial neurons to the contralateral cortex

(A-D) pCIG+pCAG and pCIG-miR-495+pCAG were electroporated into the mouse foetal brain at E13.5, and the brain sections in P9 were stained with DAPI. (A, C) is the electric lateral cortex; (B, D) is the contralateral cortex; (A', B') are enlargements of the solid white boxes of (A, B), respectively; (C, D') are enlargements of the solid white boxes of c and d, respectively; and red arrows indicate the distribution of projection fibres. Scale bar: 500 μm.

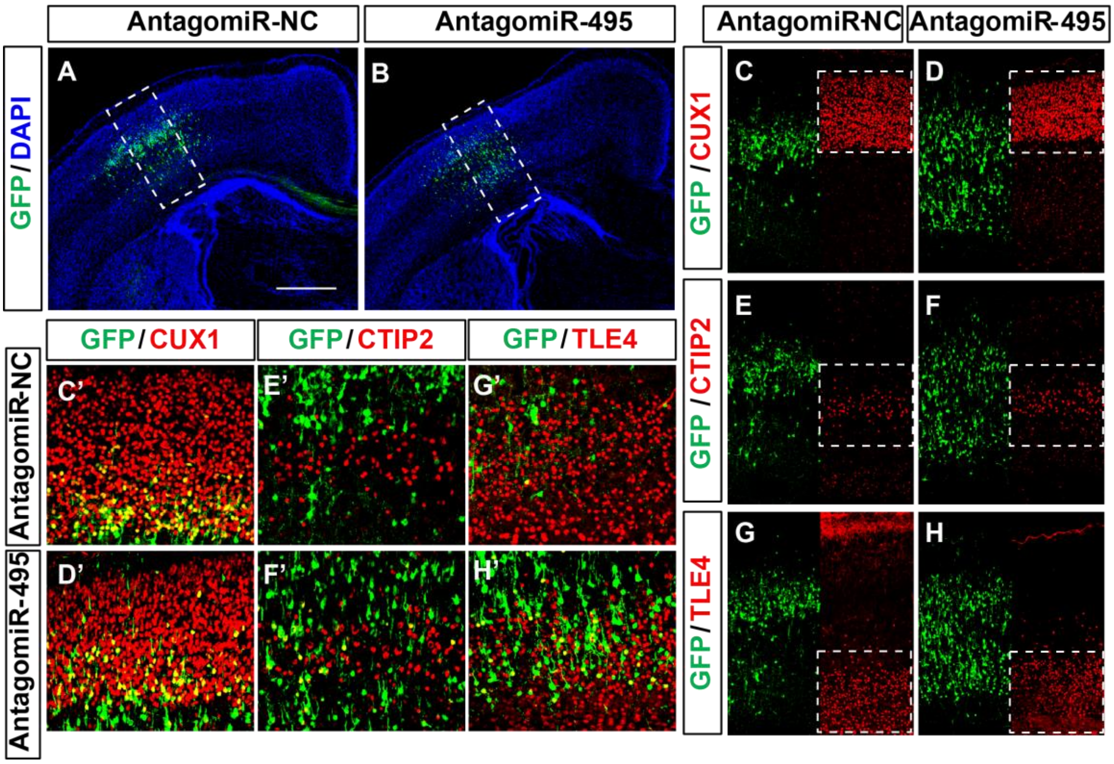

Figure S4 Knockdown of miR-495 with antagomiR promotes deep-layer neuron generation

(A-H) AntagomiR-NC+ pCAG and AntagomiR-495+pCAG were electroporated into the mouse embryonic brain at E13.5, and the brain sections at P3 were immunostained with cortex markers (CUX1, Ctip2, and TLE4). (C'-H') are enlargements of the solid white boxes of (c-h), respectively. Scale bar: 500 μm.

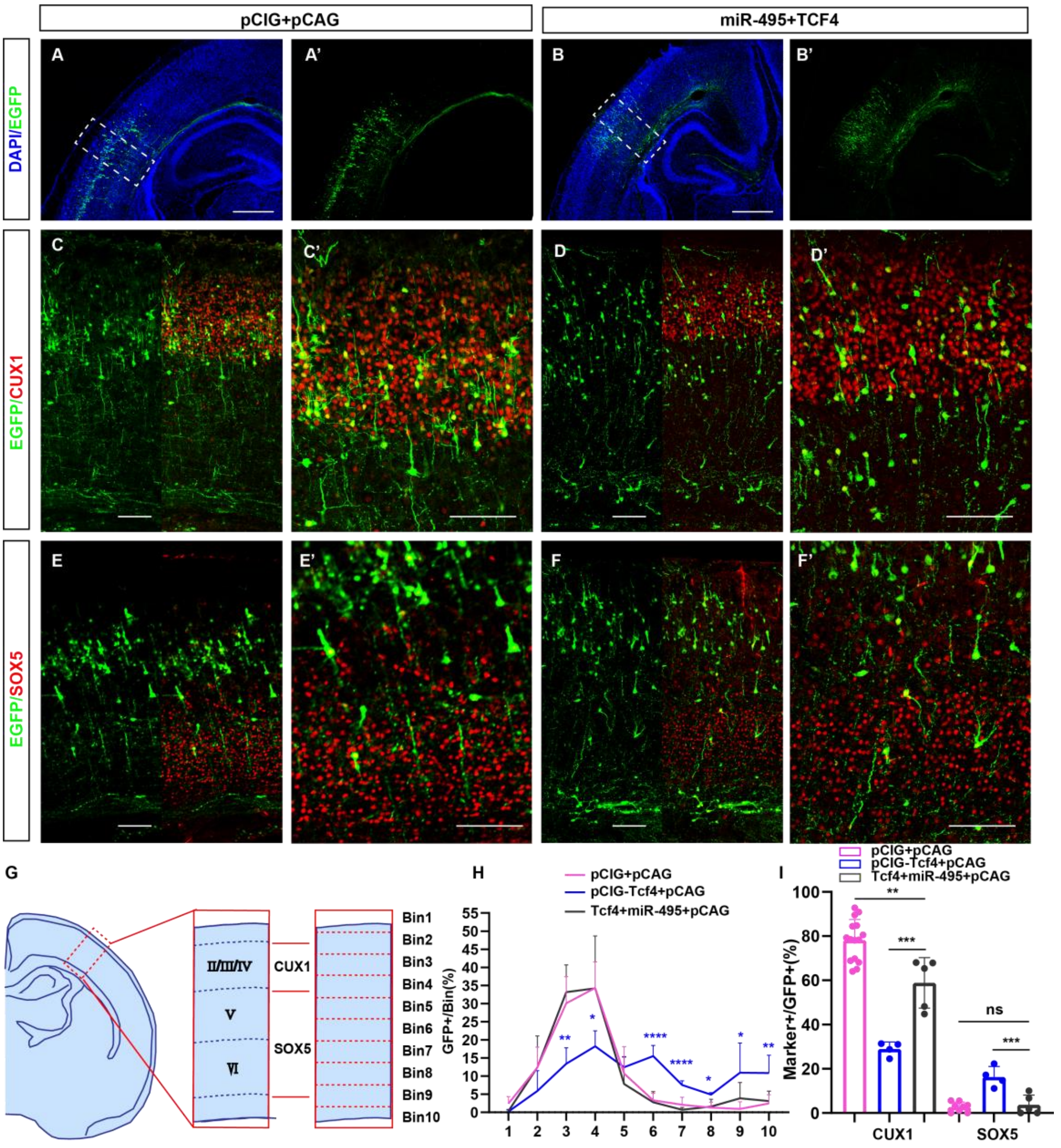

Figure S5 TCF4 rescues miR-495-determined neuronal fate

(A, A', C, C', D, D') pCIG+pCAG were electroporated into the mouse brain at E13.5, and the brain sections in P3 were stained with DAPI/EGFP/CUX1/SOX5. (B, B', D, D', F, F') pCIG-miR-495+TCF4 were electroporated into the mouse brain at E13.5, and the brain sections in P3 were stained with DAPI/EGFP/CUX1/SOX5. (G) Schematic diagram of the cerebral cortex division bin1-bin10. (H) We divided coronal sections of the cerebral cortex into 10 bins and calculated the distribution of EGFP+ cells

in the cortex. (pCIG+pCAG: n=15 sections from 13 brains, TCF4+pCAG: n=4, TCF4+miR-495+pCAG: n=5). (I) Statistical analysis of the percentage of CUX1+/GFP+ cells and SOX5+/GFP+ cells (pCIG+pCAG: n=15 sections from 13 brains, TCF4+pCAG: n=4, TCF4+miR-495+pCAG: n=5). Scale bar: 500 μm (A, B) and 100 μm (C-F, C'-F'). Statistical significance was determined using one-way ANOVA. Results are expressed as the mean ±SD. P values are shown as *P<0.05, **P<0.01, ***P<0.001.

Table S1 The primer used in the clone

Gene symbol	Primer sequences	purpose
miR-495	5'-CCGCTCGAGGCAAGGATGGTAGGCAACAT -3'<br>5'- CCGGAATTCTTTCTGGGCTTCTCTTATCTGAA -3'	Over-expression
TCF4	5'-CCGCTCGAGGGGAACACTCATGTGAGACAC- 3'<br>5'-CGGAATTCAAACGGGGTTAAGGAGCAGT - 3'	Over-expression
miR-495	5'-GTCGTATCCAGTGCAGGGTCCGAGGTATTCGCACTGGATACGACAAGAAG -3'	RT-PCR
miR-495*	5'-GTCGTATCCAGTGCAGGGTCCGAGGTATTCGCACTGGATACGACCGAAAA -3'	RT-PCR
U6	5'-GTCGTATCCAGTGCAGGGTCCGAGGTATTCGCACTGGATACGACAAAATATG-3'	RT-PCR
miR-495-up	5'- AAACAAACATGGTGCACTTCTT -3'	Real-time PCR
miR-495*-up	5'- GAAGTTGCCCATGTTATTTTTCG-3'	Real-time PCR
U6	5'-GCGCGTCGTGAAGCGTTC-3'	Real-time PCR
mir-495Sponge-A	5'-GTAACCAAGAAGTGCTGTGTTTGTTATTTTTTTAAGAAGTGCTGTGTTTGTTAG -3'<br>5'-GTTACCTAACAAACACAGCACTTCTTAAAAAAATAACAAACACAGCACTTCTTG -3'	miR-495SP
mir-495Sponge-B	5'-GTAACCAAGAAGTGCGATGTTTGTTATTTTTTTAAGAAGTGCGATGTTTGTTAG-3'<br>5'-GTTACCTAACAAACATCGCACTTCTTAAAAAAATAACAAACATCGCACTTCTTG-3'	miR-495SP

sh1-Tcf4	5′-GCCTCGTCATCTCCCAATTAT-3’,	Knockdown
Sh2-Tcf4	5′-GCCTCGTCATCTCCCAATTAT-3’,	Knockdown
Sh3-Tcf4	5′-CCCAGTACTATCAGTATTCAA-3′	Knockdown
Pak3	5’- GAGCTGTGAAATCAGCTGCAACTGAAAATGTCTGA-3’	Probe
	5’ - CTAACGGCTACTGTTCTTAATTGCTTCCTTTGCGG -3’	
Tcf4	5’-CCGTCCAGGAACTATGGAGA-3’	Probe
	5’ -CTGTTGTTCGTGTGGTCAGG -3’	
Pbrm1	5’-ACATGACTCCAATGCAGCAG-3’	Probe
	5’ -GCCCGTTCCAATACTTCAAA -3’	
E2f2	5’-ACAACATCCAGTGGGTAGGC-3’	Probe
	5’ -CCACAGGTATTCGTCCTGGT -3’	
Zfp36l1	5’-CGAGACCTCTCCGCTGAC-3’	Probe
	5’ -CAAGGTAGGGGAGTCTGAGC -3’	
Pak3	5’-AACTGCAAGCCTTACCCCTCA-3’	Dual-Luciferase
	5’-AACATTGATGTTTGTCGTTTTATTTG -3’	
Pbrm1	5’-TCACATCACTGTTTCTTCTGTGGAA-3’	Dual-Luciferase
	5’-TCACTTTGCAAACTGTTGTTTTTATT-3’	
Bmi1	5’-GACTGTTAAGGAAAAGATTTTCAACC-3’	Dual-Luciferase
	5’-CATCTTTCTGTTGTTTTATTAAAAGACAA-3’	
Msx1	5’- GTGGGTCCAG AGTCACCTC-3’	Dual-Luciferase
	5’- CTTGTAGCTTAAAATTAATTTATTTAACAAATATAGC-3’	
Dnmt3a	5’- GGACATGGGGGCAAACTGAA-3’	Dual-Luciferase
	5’- GCGGAAGCTGATGTCTTTGC-3’	
Onecut1	5’-CCGCTCGAGACCACGGACTAGTACCTCGG-3’	Dual-Luciferase
	5’-GCTCTAGAGGCATTTTAGTGAATCTCAGTG-3’	
Ddx3x	5’-GCCTGCTCTGTAGTAGGTCACCC -3’	Dual-Luciferase
	5’-TGCAGTTCAACTTTTTATTTTAATAAAACC-3’	
Igf1	5’-AGGAAGTGCAGGAAACAAGACC-3’	Dual-Luciferase
	5’-ACCAGTTAATCAAACATGATTAATTTTAAT-3’	
Tcf4	5’-GTCCAAGTTGCTACCTTGCTTCA -3’	Dual-Luciferase
	5’-CTATGTGCACGAGAGGTGAAATG-3’	
Elavl4	5’-ATTTCTCACCCTTACTTATTAAAATATATATATAAA-3’	Dual-Luciferase
	5’-TTCATTCTACACACTTTCATTTATTGTCT-3’	
E2f2	5’-AGCGTCCTGCATCTGTCTACCT -3’	Dual-Luciferase

	5’-ACCAGAGCCGCCATTATTTAAT-3’	
Hey1	5’-CGGTTGTCAACACCACCCTA-3’	Dual-Luciferase
	5’-CCAAGTGCAGGCAAGGTCTA-3’	
Nedd1	5’-AGACTCTGGGCACCTTGATT -3’	Dual-Luciferase
	5’-AGGCCACAGAAACTTGACACA-3’	
Nfib	5’-GTACAGAGGCCTAGGGCAAC-3’	Dual-Luciferase
	5’-TCTGCCAGCAAGACTGTAGC-3’	
Ctip2	5’-GCGCATACGTGGGGGACA -3’	Dual-Luciferase
	5’-TCAAGTTAAATGTCCCAATTTATTTTC-3’	
Ctnnd2	5’-CAGGACACGAGGCACTCC-3’	Dual-Luciferase
	5’-TGGCGTCCAAGAATGTGTGA-3’	
Cdc6	5’-GTCCGACTTGTTTGGGAGGT -3’	Dual-Luciferase
	5’-TCTTTGCCACTACGCCATGT-3’	
Sel1l	5’-CGGTGACCACTGGGGAGCTG-3’	Dual-Luciferase
	5’-CGTCACCAGCCGTTATACTGCAT-3’	
Igf1r	5’-TCCTCGGACACACCGAAGC -3’	Dual-Luciferase
	5’-GCATACAGAATTCTTTTATTTAACTTAATCC-3’	
Pbx3	5’-GGCGCTTTCCCAGCTGACAT-3’	Dual-Luciferase
	5’-CAATCCAGGGTGTGAGCCAGT -3’	
Cdh1	5’-GTGTGGCACCATGGGAGAT -3’	Dual-Luciferase
	5’-ACAGTTTAACAAAACTCTTTAATAAAATTCATAA-3’	
Zfp36l1	5’- GCCAGGGTAGGGAGGGACC-3’	Dual-Luciferase
	5’-GAAAAACGGGGTTTATTGATTTTT-3’	
Cdk6	5’-CATCTGAACACATTGGCGGC -3’	Dual-Luciferase
	5’- GTGCTGGTGGTTGAGAGCTT-3’	
Rybp	5’-TGCCCTGCTATAGTACTCCGT-3’	Dual-Luciferase
	5’-TAACAGTCGTGCACATGCCA-3’	
Sox5	5’-ACTTGAAGAAGCCCTGTCCG -3’	Dual-Luciferase
	5’- GCCCCCAAACAGAACAGAAC-3’	
Hipk1	5’-CTGCTGAATGTGTATGCGCC-3’	Dual-Luciferase
	5’-TGCATTGCAACTGCTCTACTT-3’	
Neurod6	5’- TCTCTCACTATGCAAGATGAATTAAATGC -3’	Dual-Luciferase
	5’- TTTGTAAGTGGAAATATCTATGTTTAATTGCT-3’	
Mll1	5’- GGTCACCCACTCCATTAGGC-3’	Dual-Luciferase

	5’- GGTGAGCTGGGTCCTGAAAA -3’	
Lhx2	5’- TGACTCGCCACCCCCTTCT -3’	Dual-Luciferase
	5’- AGTCTTTTCGATAAGTGATTTTTATTACTGGT-3’	
Elavl1	5’-GCAGATGTTTGGCCCCTTTG-3’	Dual-Luciferase
	5’- GCCAGTAACTGCACTAGCCT-3’	
Rgma	5’-CTGCCCTTTCACTTGTCTCCTAG -3’	Dual-Luciferase
	5’-TAAAAGAAAAATAACAAAACAAAACCAACTTTACTT-3’	
Foxo1	5’- CAGGAACTGAGGAGCAGTCC-3’	Dual-Luciferase
	5’- GTACTTAGGCGCACAGAGCA-3’	
Cxcr4	5’- GGACACTCTTCCGTCTCCAC -3’	Dual-Luciferase
	5’- ACATTTGTAGGGAGTGAAATCAA-3’	
Pou3f1	5’- CGGCCTGGACTCTTTTTGTTG-3’	Dual-Luciferase
	5’-TCCTGGGGTACATGTTTATGTGA-3’	
Ctip1	5’-ATCACACACCGCTCTTCAGG -3’	Dual-Luciferase
	5’-CAGGTTAATGCAGACAACTGCC-3’	
Sox13	5’-CATTCCCAAGATGGGGGTCC-3’	Dual-Luciferase
	5’-TGGTCGGTCTTTATTACACCCA-3’	
Rorb	5’- GTGCTGCGGTCTGCAAAT -3’	Dual-Luciferase
	5’- GTGGCTTCAAATCCTTTTGG-3’	
Meis2	5’- AAGTTGGGCAGCTTTCCTCA-3’	Dual-Luciferase
	5’- AAGCTTAGAATTCCCAACTCCA-3’	
Tgfb2	5’-GCCAGGACACGAAAATCACG -3’	Dual-Luciferase
	5’-TGCTGGCTTCTAGACCCGT-3’	
Meis1	5’- CAAAGCATTGGTCATGTGTGTAT-3’	Dual-Luciferase
	5’- CTGGACCTGGAGTTTGCATAC-3’	
Dmd	5’- TGGCAGATGATTTGGGCAGA-3’	Dual-Luciferase
	5’-CGGTAGTCTCCTGGCTTTGG-3’	
Sp5	5’- GGACACTTTCGAGGCCACTC-3’	Dual-Luciferase
	5’- CGAGGAGACCCTGGAATGAAG-3’	
Phf6	5’-GCTGGTAGAACAGCGTTTTG -3’	Dual-Luciferase
	5’-CCCCAGGAACAAAGAGGTC-3’	
Fmr1	5’-ATAAGCTACATAATTCCGAAGTTATATTTCCTCTA-3’	Dual-Luciferase
	5’-TAACCTGCTTTCAATGTTTCTCAGAC-3’	
Pak7	5’-GAGGATTCACACAGGATGCAAAGCT -3’	Dual-Luciferase

	5’-CAAACACTTACTTTATTTGTGGTGCAGC-3’	
Foxp1	5’- GGGAAGAAAGAAAAACTCCACACA -3’<br>5’- GTCAGGAGGGTATGGCACTG -3’	Dual-Luciferase
Foxg1	5’- GGGGGACCAGACTGTAAGTG-3’<br>5’-ACACGGGCATATGACCACAG-3’	Dual-Luciferase
Sox11	5’-GGTGTCTCAGCATCCAACCA-3’<br>5’-CCAGAGAGCTGTCCAACACA-3’	Dual-Luciferase
Onecut2	5’- CCCACCCCTCAGCTGTATTT -3’<br>5’- AAGATCCCAGTGACAGCAGC-3’	Dual-Luciferase
Cdkn1b	5’-AATTAAGAATATTTCCTTGTTTATTAGATACATCA -3’<br>5’- GCACTCAATAAATAACTACGGAAGTTTTC-3’	Dual-Luciferase
Satb1	5’-ACAGCCAGCACTCAAGGTTT -3’<br>5’- CAAGCCCCTCCCCTAAACTG-3’	Dual-Luciferase
Dll1	5’-GATGGAAGCGATGTGGCAAAATT-3’<br>5’-TGAATTTCTTCATTAACAAAACAGTAAAAAACTC-3’	Dual-Luciferase
Satb2	5’-TGCAACCTTGTCAAAGACCTC -3’<br>5’- TTCTAACAGCCTAACAATGCACA-3’	Dual-Luciferase
Nufip2	5’- TGTGGTAGGCCAGTTTCAGA -3’<br>5’- CAAGCCTTGGACAGAGAAGG-3’	Dual-Luciferase
Tcf12	5’- CAGAGTCATCAGTAGGCTAAATAGAAG-3’<br>5’- AAGGATGGCACATTTATTGCTACATA-3’	Dual-Luciferase

Table S2 The microarray raw data

Time	mmu-miR-92b-3p	mmu-miR-92b-5p	mmu-miR-495-3p	mmu-miR-495-5p	mmu-miR-494-3p	mmu-miR-667-3p
E12.5-1	9,665	541	3,213	23	2,610	650
E12.5-2	10,143	307	2,946	55	2,909	698
E12.5-3	7,749	257	2,636	16	3,290	420
E14.5-1	6,273	186	1,242	7	2,723	302
E14.5-2	7,205	335	2,029	3	4,158	199
E14.5-3	8,607	212	2,296	0	4,607	235
E16.5-1	2,740	114	1,988	59	1,415	203
E16.5-2	3,184	119	1,148	5	1,335	209
E16.5-3	4,548	127	2,120	5	1,422	231
E18.5-1	2,764	76	1,863	10	1,548	168
E18.5-2	2,490	123	2,706	16	1,600	224
E18.5-3	2,276	84	1,895	5	1,626	203
